# Massive experimental quantification allows interpretable deep learning of protein aggregation

**DOI:** 10.1126/sciadv.adt5111

**Published:** 2025-04-30

**Authors:** Mike Thompson, Mariano Martín, Trinidad Sanmartín Olmo, Chandana Rajesh, Peter K. Koo, Benedetta Bolognesi, Ben Lehner

**Affiliations:** ^1^ Centre for Genomic Regulation (CRG), Barcelona Institute of Science and Technology, Dr. Aiguader 88, Barcelona 08003, Spain.; ^2^ Institute for Bioengineering of Catalonia (IBEC), Barcelona Institute of Science and Technology, Barcelona 08028, Spain.; ^3^ Simons Center for Quantitative Biology, Cold Spring Harbor Laboratory, Cold Spring Harbor, NY 11724, USA.; ^4^ Universitat Pompeu Fabra (UPF), Barcelona 08002, Spain.; ^5^ ICREA, Pg. Lluis Companys 23, Barcelona 08010, Spain.; ^6^ Wellcome Sanger Institute, Wellcome Genome Campus, Hinxton CB10 1RQ, UK.

## Abstract

Protein aggregation is a pathological hallmark of more than 50 human diseases and a major problem for biotechnology. Methods have been proposed to predict aggregation from sequence, but these have been trained and evaluated on small and biased experimental datasets. Here we directly address this data shortage by experimentally quantifying the aggregation of >100,000 protein sequences. This unprecedented dataset reveals the limited performance of existing computational methods and allows us to train CANYA, a convolution-attention hybrid neural network that accurately predicts aggregation from sequence. We adapt genomic neural network interpretability analyses to reveal CANYA’s decision-making process and learned grammar. Our results illustrate the power of massive experimental analysis of random sequence-spaces and provide an interpretable and robust neural network model to predict aggregation.

## Teaser

xAI trained on >100,000 random peptides predicts and elucidates primary sequence determinants of aggregation.

## INTRODUCTION

**Fig. 1.**
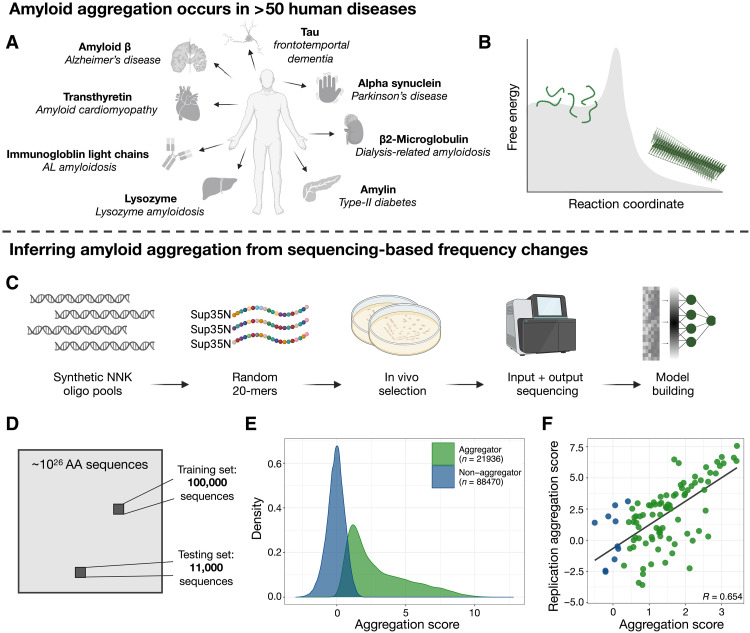
Quantifying the aggregation of >100,000 random peptides. (**A**) Examples of amyloids in human diseases. (**B**) The amyloid state is thermodynamically favorable but requires overcoming a kinetic barrier. (**C**) Experimental design. (**D**) While we explore more than 110,000 sequences, our dataset is a tiny sample of the possible sequence space. (**E**) The assayed aggregation scores of sequences labeled “aggregators” and “non-aggregators” in our experiment. (**F**) An example of a follow-up replication experiment using a synthesized library (NNK3; see fig. S2 for others; data file S3). AA, amino acid.

Specific insoluble protein aggregates in the form of amyloid fibrils characterize more than 50 clinical conditions affecting more than half a billion people (Fig. 1A) (*1*). These include common neurodegenerative disorders and the most frequent forms of dementia. Nonetheless, amyloids are present in all kingdoms of life and can have functional roles, including in humans (*2*). Protein aggregation is also a major problem in biotechnology, for example, in the production of enzymes, antibodies and other protein therapeutics (*3*). The importance of amyloids across biological functions and diseases has spurred massive research efforts, yet the determinants and mechanisms of their formation remain quite poorly understood (*4*, *5*).

Recent advances in cryogenic electron microscopy have allowed the atomic structures of many mature amyloid fibrils to be determined (*6*). Amyloids share a cross-β structure wherein hydrogen-bonded β strands are perpendicularly stacked along the fibril axis, creating β sheets that face each other and are parallel to the fibril axis (*4*, *7*, *8*). Amyloid fibrils of human proteins typically have hydrophobic cores, and hydrophobicity and β strand propensity form the basis of many computational methods to predict amyloid propensity from sequence (*9*–*15*). However, other amyloids, for example yeast prions, have very different sequence composition, hinting at a richer diversity of amyloid-forming sequences (*16*, *17*).

In contrast to the remarkable advances in the structural characterization of mature fibrils, the process of amyloid formation is much less understood. Time-resolved structure determination has been used to study the in vitro assembly of amyloids, revealing a notable diversity of intermediate structures appearing and disappearing as fibrillation proceeds (*18*, *19*). However, how this process initiates and why it only occurs for some sequences under physiological conditions remains unclear. Mature amyloid fibrils are very stable and are likely to be the thermodynamically favored state at high protein concentration for many proteins (*20*, *21*). There is, however, a very high energy barrier to amyloid nucleation for most proteins, i.e., the process is under kinetic control (Fig. 1B) (*21*). The kinetic control of amyloid nucleation is, therefore, the key problem to understand: What are the sequence-level determinants that cause some peptides to nucleate amyloid formation on timescales relevant to biology?

We believe that our ability to understand and predict amyloid formation is currently data-limited. To date, computational methods to predict aggregation have been trained and benchmarked on very small and biased experimental datasets. For example, several of these methods are trained on datasets that can be trivially explained by hydrophobicity or sequence length alone (fig. S1 and table S1), making it unlikely that they have learned representations of aggregation that generalize across the diversity of sequence space (*12*–*15*, *22*–*25*). More explicitly, even for a peptide composed of just 20 amino acids there are 20^20^ (>10^26^) different sequences that this peptide can realize. Such a large sequence space is unlikely to have been accurately modeled by methods trained on tens or a few hundred sequences.

To directly address this data gap, we have developed a massively parallel selection assay that allows the aggregation of thousands of different proteins to be tested and quantified in a single experiment (*26*, *27*). This has allowed us to quantify the change in aggregation rates for all possible substitutions, insertions, and deletions in the amyloid-β peptide that aggregates as a hallmark of Alzheimer’s disease. The resulting measurements agree very well with in vitro nucleation kinetic rate constants (*26*, *27*). However, these datasets are limited to testing the effects of small changes to a single sequence, hindering utility for general-purpose model building.

Here, we apply this approach at a much larger scale and quantify the aggregation of >100,000 peptides with completely random sequences. We use the resulting massive dataset to evaluate existing aggregation prediction methods and find that unlike their performance on previous, potentially biased datasets, they are only moderately predictive across a much wider sequence space. We therefore use the data to train CANYA, a convolution-attention hybrid neural network. This fast model dramatically outperforms existing predictors of protein aggregation when tested on >10,000 additional sequences, demonstrating the power of massive experimental sequence-space exploration. Subsequent post hoc explainable artificial intelligence (xAI) analyses provide mechanistic insights into CANYA’s decision-making process and learned grammar. CANYA provides a robust and interpretable neural network model for understanding and predicting amyloid-forming proteins. More generally, our results not only provide a very large and well-calibrated dataset to train and evaluate models beyond CANYA, but they also demonstrate the utility of massive experimental analysis of random protein sequence spaces.

## RESULTS

### Massively parallel quantification of the aggregation of >100,000 sequences

To better understand the sequence determinants of peptide aggregation, we used an in-cell selection assay to quantify the rate of aggregation of more than a hundred thousand peptides with fully random sequences. We generated four libraries (NNK1 to 4) of random 20 amino acid peptides using NNK degenerate codons (where N = A/C/G/T and K = G/T) and expressing them as fusions to the nucleation domain of Sup35 (Sup35N), a yeast prion–forming protein that allows fitness-based selection for amyloid aggregation (Fig. 1C) (*26*–*28*). Briefly, fusion sequences that nucleate amyloids sequester Sup35, resulting in translational readthrough of a premature stop codon in the *ade1* gene so that cells containing those sequences can grow in medium lacking adenine. Enrichment or depletion of each sequence after selection is quantified by deep sequencing, with, at least for amyloid-β variants, enrichment scores linearly related to the log of in vitro amyloid nucleation rates (*26*, *27*, *29*).

Each library was selected independently, and sequencing was used to quantify the relative enrichment (“aggregation score”) for each genotype in the library. Sequences in the first three experiments made up our training and testing sets (NNK1–3, *N* = ~111,000; Fig. 1D and data files S1 and S2), corresponding to about a 1/10^21^ fraction of the possible sequence space (20^20^), while sequences from the fourth experiment (NNK4, *N* = ~7000) were used as a held-out test dataset. After data processing and quality control, the vast majority of sequences had an aggregation score of 0. Consequently, we classified sequences with an aggregation score significantly greater than 0 [one-sided *Z* test, false discovery rate (FDR)–adjusted *P* value ≤0.05] as aggregators (*n* = 21,936) and all other sequences as non-aggregators (*n* = 88,470) (Fig. 1E). These aggregation scores are reproducible, as measured by an additional selection experiment on a designed library (replication library) requantifying the aggregation of 400 sequences sampled across all four libraries (Pearson correlation range 0.506 to 0.797; Fig. 1F and fig. S2).

### Aggregating sequences span a large sequence space and are poorly predicted by existing computational methods

**Fig. 2.**
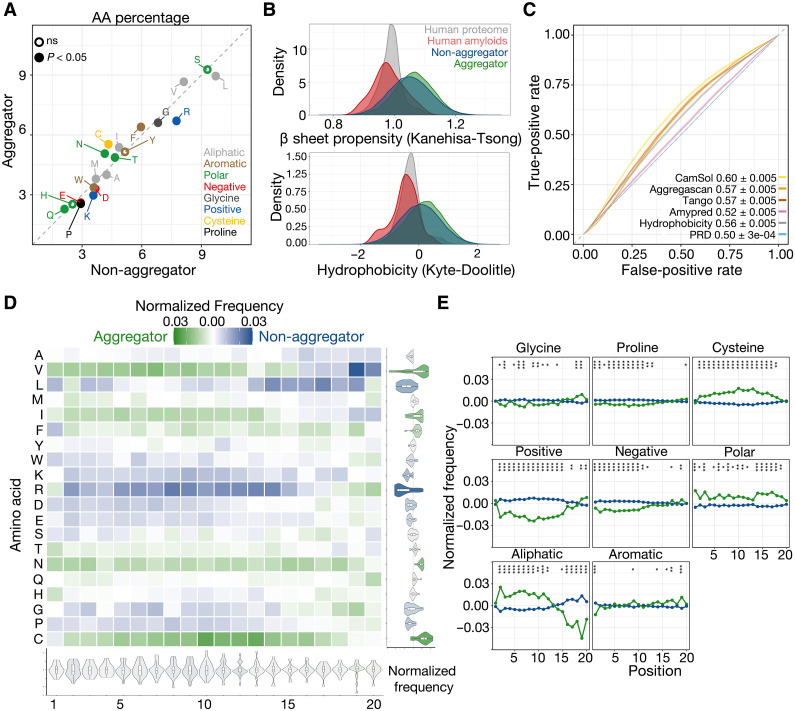
Aggregation is poorly predicted by existing models and subtly related to amino acid composition. (**A**) The percent composition of residues grouped by their physicochemical properties in aggregators and non-aggregators. (**B**) The hydrophobicity and β sheet propensity of assayed sequences relative to known human amyloids (table S3) and the human proteome. (**C**) The predictive power (AUROC ±95% CI) of previous amyloid predictors on the random sequences. (**D** and **E**) The position-specific differences in amino acid frequencies across aggregating and non-aggregating sequences. Asterisks indicate marginal *P* value (chi-square test) lower than 0.05 “*”; lower than 0.01 “**”; lower than 0.001 “***.”

After classifying sequences as aggregators and non-aggregators, we sought to characterize each class through amino acid composition (Fig. 2A), physicochemical properties (Fig. 2B), and current amyloid prediction tools (Fig. 2C). First, we examined the differences in amino acid frequency between aggregating and non-aggregating sequences. Differences in frequencies were generally modest; however, we observed statistically significant differences owing to the large sample size of our data. When looking at composition independent of position, aggregators had higher frequencies of cysteine (difference in frequency 0.012, *P* < 2 × 10^−16^), asparagine (0.009, *P* < 2 × 10^−16^), and isoleucine (0.005, *P* < 2 × 10^−16^), and lower frequencies of arginine (−0.010, *P* < 2 × 10^−16^), leucine (−0.008, *P* < 2 × 10^−16^), and lysine (−0.006, *P* < 2 × 10^−16^; Fig. 2A, see table S2 for full differences). Moreover, both aggregators and non-aggregators covered the β sheet propensity and hydrophobicity spaces of the human proteome and known amyloid sequences, and aggregators had slightly higher values of both than non-aggregators on average (difference in means of hydrophobicity = 0.130, β sheet propensity = 0.012, both two-way *t* test *P* values <2 × 10^−16^; Fig. 2B). Considering position-specific composition, differences were again modest, ranging from a difference in frequency from −0.06 to 0.03 (Fig. 2D). Subsequently, we grouped amino acids by their physicochemical properties to check for broader, position-specific differences between the two sequence classes (Fig. 2E). Toward the N terminus of the random sequence (i.e., closer to Sup35N), aggregators were significantly enriched (chi-squared test) for aliphatic residues (min. *P* value = 1.54 × 10^−13^, position 2 difference = 0.033), and significantly depleted for positive (min. *P* value = 1.57 × 10^−25^, position 9 difference = −0.032) and negative residues (min. *P* value = 3.14 × 10^−11^, position 2 difference = −0.016). The differences in charge waned toward the C terminus (min. *P* value above position 15 = 1.03 × 10^−3^, position 20 charged difference = 0.011), however, and frequency differences in aliphatic residues changed such that aggregators were significantly depleted for aliphatic residues relative to non-aggregators (min. *P* value = 5.77 × 10^−39^, position 19 difference = −0.058). Several groupings showed other position-sensitive differences, such as an enrichment of aromatic residues toward the C terminus in aggregators (min. *P* value = 5.09 × 10^−6^, position 19 difference = 0.015), an enrichment of varying strength for polar residues in aggregators (*P* value = 5.57 × 10^−8^ position 1 difference = 0.023, *P* value = 9.41 × 10^−7^ position 17 difference = 0.020), and the enrichment of cysteines away from the ends of the random construct (min. *P* value = 1.11 × 10^−28^, position 10 difference = 0.023).

Despite statistical significance, we highlight that differences in sequence space are subtle. In other words, the collection of slight variation in amino acid frequencies offers minimal insight or definitive conclusions around the overall properties or characteristics determining aggregation in our experiment. To attempt to elucidate characteristics that separate the sequence classes and consequently learn important axes of variability, we turned to dimensionality reduction techniques. In addition to manually examining differences within the first several dimensions, we also used the scores in lower-dimensional space as features in a logistic multiple regression task to distinguish aggregators from non-aggregators. Using principal components analysis (PCA), we observed no clear separation between aggregators and non-aggregators whether we used amino acid composition alone {cumulative variance explained from the top 10 principal components (PCs)s = 54.7%, area under receiver operating characteristic curve (AUROC) using all 10 PC scores = 0.601, 95% confidence interval (CI) = [0.596, 0.607]; fig. S3}, or maintained positionality of the amino acids when fitting the model (cumulative variance explained from the top 10 PCs = 3.1%, AUROC = 0.564, 95% CI = [0.559, 0.570]; fig. S3). This modest separation between classes of sequences was consistent even when using nonlinear embedding techniques (first 10 Uniform Manifold Approximation and Projections AUROC = 0.580, 95% CI [0.575, 0.586]), or adding amino acid propensities to the dimensionality reduction tools (first 10 PCs AUROC = 0.614, 95% CI [0.608, 0.619]; fig. S3).

As dimensionality reduction methods were unable to distinguish the classes of sequences, we next explored whether separation is possible using existing amyloid predictors. Beyond hydrophobicity indices, several of these methods include structural information (*30*) or model biophysical mechanisms (*12*), potentially enabling them to capture more complex features of aggregation. We applied several state-of-the-art amyloid prediction algorithms to our data and found that the methods either failed to generalize to our data or had only modest predictive power (Fig. 2C, CamSol, highest AUROC = 0.598, 95% CI [0.593, 0.603]). We posit that, since many of these tools have been trained on very small sets of known amyloids or moderate numbers of short hexamer sequences, their applicability to our experimental data may be limited. To understand where the methods underperformed, we examined the scores from the highest performing methods [CamSol (*13*) and TANGO (*12*)] and found that non-aggregating sequences with a high-predicted aggregation score had higher hydrophobicity (two-sided *t* test *P* value <2 × 10^−16^) than all other non-aggregating sequences (table S4). We also found that low-predicted aggregators had higher presence of positive (two-sided *t* test *P* value <2 × 10^−16^) and negative (*P* value <2 × 10^−16^) residues than all other aggregators (table S5).

### CANYA: A hybrid neural-network that accurately predicts amyloid aggregation

**Fig. 3.**
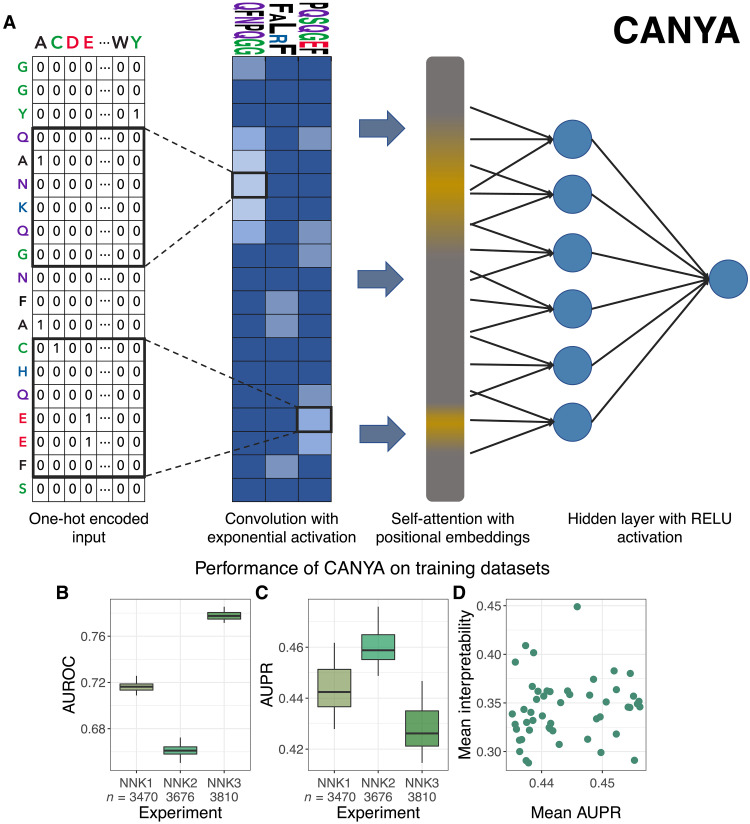
CANYA. (**A**) CANYA is a three-layer neural network with 17,491 parameters. The model contains 100 filters, a single attention head with key length 6, a dense layer with 64 nodes, and finally a sigmoid output layer. (**B** to **D**) Evaluation metrics across the top 50 performing (of 100) model fits of CANYA. (B) The AUROC for held-out testing sequences. (C) The AUPR for held-out testing sequences. (D) The interpretability score (KL divergence; Methods) calculated on all held-out test sequences plotted against the mean AUPR across experiments. See fig. S4 for results on all 100 model fits.

Given that previous approaches failed to accurately predict aggregation status within our dataset, we built our own model to capture the sequence-aggregation score landscape. Concretely, we developed a hybrid neural network that we term CANYA (for Convolution Attention Network for amYloid Aggregation). Although a neural network may seem inherently less interpretable than simpler models, as we explain below, the architecture of CANYA is not only simple but also biologically motivated. CANYA builds off the observation that known amyloids are composed of interacting short sequences, such as stacked β sheets, and treats this information as an inductive bias for the model—first, the sequences are passed through a convolutional layer that finds “motifs,” then these motifs are passed through an attention layer to learn positional effects of motifs and to encourage these motifs to interact with each other (Fig. 3A). Moreover, we set the filter lengths of the convolutional layer based on the distribution of secondary structure lengths in 80 known amyloid fibril structures [WALTZ-DB (*31*); fig. S5]. Though—to our knowledge—this class of models may be less commonly used with proteins, convolution-attention hybrid models have been used in genomics and found to serve as a sound inductive bias for finding motifs and their interactions (*32*, *33*).

We trained CANYA 100 times on more than 100,000 synthetic sequences and their respective aggregation status to learn the sequence-aggregation landscape. Unlike massive, computationally intensive neural networks, CANYA comprises only three layers (spanning 17,491 parameters) and requires less than an hour to train on a basic, modern CPU. Despite this simplicity and having only observed a small fraction of the possible sequence space, CANYA substantially improved the prediction of aggregation status of held-out test sequences (average AUROC = 0.710, 0.650, 0.769 across NNK experiments 1 to 3 respectively; Fig. 3, B and C) over previous methods (max. AUROC CamSol, NNK1 = 0.617, NNK2 = 0.537, and NNK3 = 0.673). We also note that the predictive accuracy of CANYA was significantly higher than simpler linear models trained on the same dataset with amino acid composition or counts alone (fig. S6).

To understand the differences in performance across methods, we examined the sequence scores between the next best performing method (CamSol) and CANYA. We found that the largest discrepancies for non-aggregating sequences occurred in hydrophobic sequences with tryptophans, and in cysteine- or asparagine-rich sequences with few aliphatic residues in the case of aggregating sequences (tables S4 and S5). Our results not only highlight the utility of exploring a vast sequence space but also suggest that CANYA is able to contextualize physicochemical properties within sequences (e.g., among hydrophobic sequences, CANYA adjusts its score in the presence of bulky or disruptive residues).

Crucially, we developed CANYA with the goal of interpreting the grammar of aggregation rather than maximizing predictive power. To select a model amenable to uncovering this learned grammar, we scored each trained instance of CANYA using a recently developed interpretability metric (*34*). Briefly, this metric examines the enrichment of motifs used when training the model and compares them to the set of all equal-length (*k* = 3) kmers in the training sequences (Methods). Strong enrichment (i.e., divergence from the background training sequences) indicates that a model may yield clearer resolution in downstream interpretability analyses. Though the area under the precision-recall curve (AUPR) of test sequences was more consistent than AUROC across experiments (average AUPR NNK1 = 0.434, NNK2 = 0.452, and NNK3 = 0.415; Fig. 3C), we did not find a correlation between predictive performance and this interpretability metric (correlation of average AUPR and interpretability score *r* = −0.059, *P* value = 0.6847; Fig. 3D). We therefore chose the trained model with the highest interpretability score, conditional on the fact that it scored better than the median-performant model (of 100 training runs; Methods).

### Evaluation on >7000 additional sequences

**Fig. 4.**
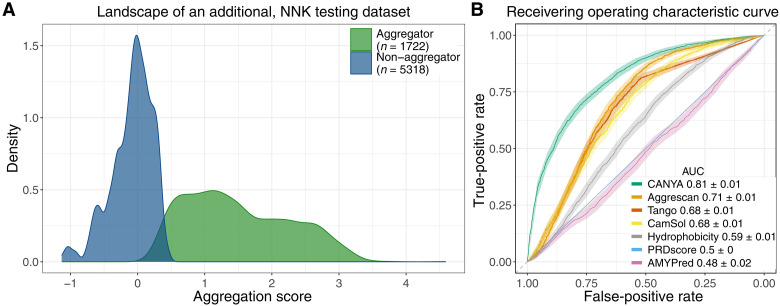
An additional experiment of >7000 random sequences. (**A**) The aggregation rates over an additional validation set of 7040 random sequences. (**B**) The predictive performance (AUROC ±95% CI) of CANYA and previous methods on the additional dataset.

To further evaluate the performance of CANYA and to compare it to that of previous methods, we quantified the aggregation of an additional ~7000 random sequences (Fig. 4A). We emphasize that the sequence spaces spanned by the training and these test sequences are effectively independent (~10^5^ and ~10^3^ samples from a >10^22^ sequence landscape). CANYA remained highly accurate on the 7000 unseen sequences (AUROC CANYA = 0.809, 95% CI [0.798, 0.821; Fig. 4B and PROC in fig. S7). Moreover, CANYA substantially outperforms all tested previous methods (*12*–*15*, *23*, *35*). The next best performing method was Aggrescan (AUROC = 0.707 95% CI [0.694, 0.719]), followed by TANGO (AUROC = 0.680 [0.667, 0.693]) and CamSol (AUROC = 0.679 [0.665, 0.693]). Neither AmyPred nor PLAAC produced significantly accurate predictors on the validation dataset, which may be indicative of overfitting on their respective training datasets—we used a simple hydrophobicity score as a baseline predictor, which scored AUROC = 0.593 (95% CI [0.579, 0.607]).

### CANYA predicts known amyloids and aggregating sequences

**Fig. 5.**
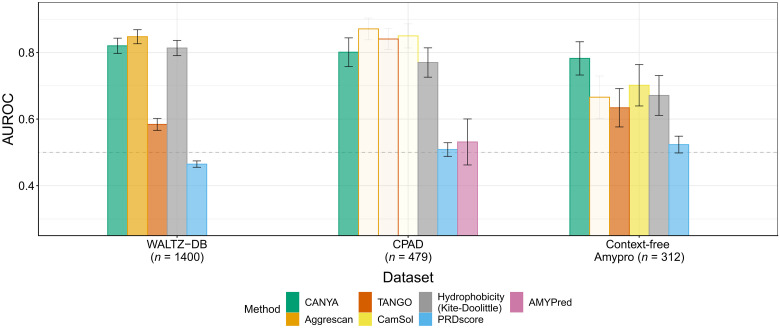
Stable performance of CANYA across diverse prediction tasks. The AUROC of CANYA and previous methods across several external datasets. Low-opacity bars represent cases in which the method used data from the testing dataset for training and thus are not valid out-of-sample evaluations. See text for additional descriptions of datasets (Methods and table S1) as well as performance reported as AUPR (fig. S7).

After establishing that CANYA can accurately predict the experimental aggregation status from primary sequence, we sought to understand whether the aggregation function learned by CANYA is applicable to different contexts or sequences with different lengths. We first considered 1400 hexapeptides from WALTZ-DB, the previously largest dataset of amyloidogenic and non-amyloidogenic sequences (*31*). Strikingly, however, on these six-amino acid peptides, no method significantly outperformed hydrophobicity for classifying aggregating from non-aggregating sequences (AUROC = 0.813 05% CI [0.791, 0.836]) (Fig. 5). The hydrophobicity distributions of amyloid and non-amyloid hexamers in WALTZ-DB are indeed very distinct (table S1), suggesting biases in this dataset or that hydrophobicity dominates the aggregation potential of such very short peptides. This cautions against the use of such short sequences for model training and evaluation.

We next considered the Curated Protein Aggregation Database (CPAD) (*36*). Although CPAD contains more than 2000 sequences, we limited our evaluation here to the 479 sequences with length >10 amino acids [median length 16 (Q1 length = 10 and Q3 length = 22], comprising 304 amyloid-forming sequences and 175 non-aggregating sequences (table S1). Several of the previous methods (including TANGO, CamSol, and Aggrescan) were directly trained on sequences within CPAD, violating the ability to evaluate their out-of-sample predictive performance on this dataset. Despite this, CANYA performed similarly as well as these methods on CPAD (AUROC = 0.801, 95% CI [0.757, 0.843], AUPR = 0.849, 95% CI [0.808, 0.884]) (Fig. 5).

Last, we evaluated whether each method could identify amyloidogenic regions of each protein in the AmyPro dataset. Specifically, we evaluated whether methods were capable of distinguishing an amyloidogenic region from a non-amyloidogenic region in the absence of any contextual region (Methods), for which we term the task “Context-Free AmyPro.” CANYA significantly outperformed all previous approaches (AUROC = 0.782, 95% CI [0.732, 0.832]; Fig. 5) and was the only method to significantly outperform hydrophobicity on this task (AUROC = 0.671, 95% CI [0.611, 0.731]).

In summary, CANYA’s performance is state of the art and consistent across diverse prediction tasks and protein sizes. We also again emphasize that by modeling motifs and their interactions, CANYA is significantly more accurate than simpler linear models that are trained over the same NNK training set (fig. S6).

### CANYA learns physicochemical aggregation motifs

**Fig. 6.**
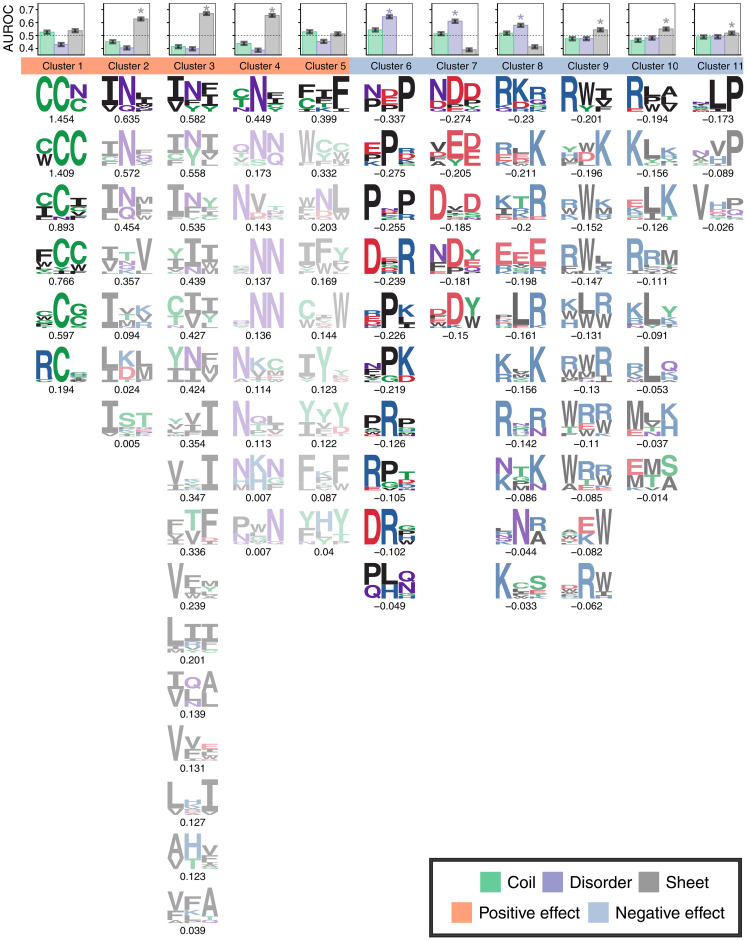
CANYA finds physicochemical aggregation motifs. The motifs found by CANYA, clustered by their physicochemical properties and GIA effect sizes, and then sorted on the basis of their effect size magnitude. Translucency represents the ratio of cluster effect size compared to the strongest cluster (Methods). The enrichment (in AUROC) of motif cluster presence in secondary structures of resolved amyloids in UniProt (Methods and fig. S8). The dashed lines represent an AUROC of 0.50, and asterisks represent structures for which the enrichment was significantly higher than both 0.50 and the second most-enriched structure.

We next performed a series of interpretability analyses to understand how CANYA assigns its aggregation score and to elucidate difficult-to-see patterns that differentiate the aggregators and non-aggregators in the training data. First, to visualize physicochemical motifs learned by the model, we constructed position-weight matrices (PWMs) using kmers that activated a given filter at least 75% of the maximum-activating kmer (Methods). We selected a filter length of 3 as this is the mode length of secondary structures in structurally resolved amyloids (Methods and fig. S5). Motifs showed clear physicochemical preferences (Fig. 6). For example, many motifs capture blocks of hydrophobicity (clusters 3 and 5), polarity (clusters 1 and 4), or charge (clusters 6 to 8). Some motifs showed heterogeneity or position-preferential effects, such as polar residues being surrounded by hydrophobic (clusters 2) or aromatic residues (clusters 3; Fig. 6).

**Fig. 7.**
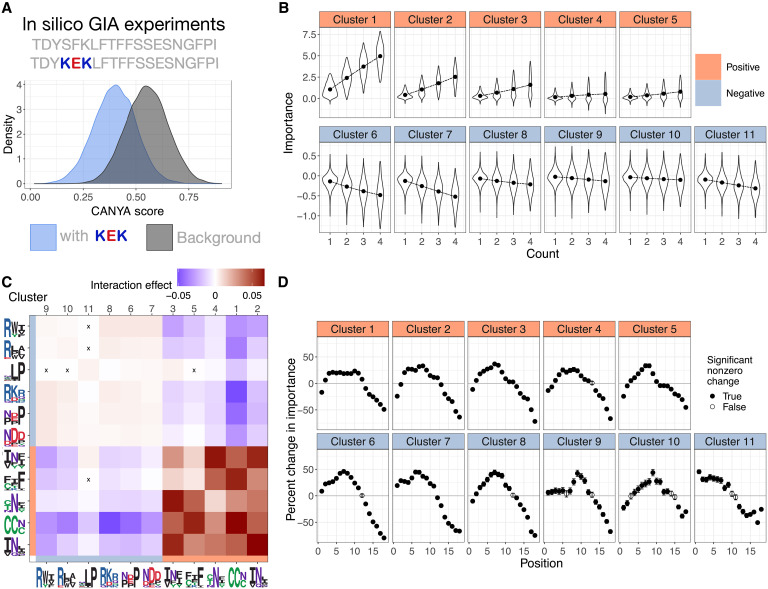
In silico experiments reveal CANYA’s learned aggregation grammar. (**A**) An example of an experiment using GIA, an explainability tool to extract importance (effect sizes) of features in a model. Briefly, model predictions for a background set of sequences are compared to predictions on the same set of sequences with a feature (motif) embedded in them. (**B**) The distribution of effects from adding one to four copies of a cluster-motif to sequences. Points represent importance. (**C**) Interaction importance from adding motifs from two clusters to sequences. Warmer colors indicate higher CANYA score than from marginally adding the motifs (and their effects) separately to sequences, whereas cooler colors represent a CANYA score lower than expected from adding marginal motif effects. “X” indicates effects that were not significantly different from 0. (**D**) The position-dependence of motif effects. Plotted is the percent change of a position-specific effect relative to the motif’s global, position-averaged effect.

We next turned to a post hoc interpretability method named Global Importance Analysis (GIA) to learn the effect of each motif (*37*). Briefly, GIA learns effect sizes by embedding a motif of interest in a set of background sequences and then comparing the difference in the model’s predicted aggregation propensity between these background sequences with and without the embedded motif (Fig. 7A). The effects learned by CANYA recapitulated previously known amyloid biology—hydrophobic motifs strongly increased a given sequence’s propensity to aggregate, and charged, proline-containing motifs lowered sequences’ propensity to aggregate (Fig. 6) (*38*–*40*). Motifs containing residues enriched in yeast prions (Q/N) typically increased amyloid propensity (motifs of clusters 1, 3, and 4), as did motifs enriched in cysteine (cluster 1) or aromatic residues (cluster 5; Fig. 6). CANYA could also uncover motifs for which specific residues had effect sizes in both directions. For example, tryptophan-containing motifs led to a negative effect when the tryptophan was surrounded by charged residues (cluster 9; Fig. 6), or a positive effect in the context of hydrophobic, polar, or other aromatics (clusters 1 and 5; Fig. 6). Notably, CANYA also distinguished hydrophobic, charged motifs with positive effect size—when the charged residue was surrounded by the hydrophobic residues (weaker motifs of clusters 2, 3, and 5; Fig. 6)—and negative effect size when the charged residue was on the outside of the motif (clusters 10, 8). These motifs further suggest that the model captures previously uncharted areas of the amyloid sequence space.

We next clustered the motifs using their amino acid similarity to generate a more concise representation of what the model has learned and to reduce the dimensionality of in silico analyses to extract further information learned by CANYA. To do so, we first generated BLOcks SUbstitution Matrix (BLOSUM) scores (which capture a similarity of amino acids based on evolutionary divergence) for each motif and then performed affinity clustering on the BLOSUM scores to derive a candidate set of clusters (Methods) (*41*). We verified that this approach results in a sound set of clusters by rerunning GIA using the clusters as the feature of interest and confirming that the learned effect size for a cluster was consistent with the motifs of which it is composed (Methods and table S6). We were left with 11 clusters on which to perform downstream in silico experiments, effectively reducing the number of experiments by at minimum one order of magnitude (from 100 filters).

### Physicochemical motif activation in known amyloid structures

We examined whether the motif clusters found by CANYA showed propensity for secondary structures in known amyloid fibril structures [from the Structural analysis of Amyloid Polymorphs (StAmP) database (*42*)]. We included in our comparison full-length resolved structures of amyloid fibrils for 114 Protein Data Bank (PDB) entries comprising amyloid structures of 23 proteins (table S7). Here, we used the activation energy of a cluster across positions to predict whether or not the corresponding position was in a β strand, other structured region (coil), or unresolved (disordered, see Methods). The AUROC from this task serves as a metric of whether high activation (high matching score) of a motif is associated with a specific structural element. Clusters with high hydrophobicity and positive effect size were most strongly associated with activating in β strands (Fig. 6 and fig. S9, max. AUROC = 0.669, 95% CI [0.659, 0.680], cluster 3), whereas the strongest enrichment among negative-importance clusters was observed in disordered regions (max. AUROC = 0.645, 95% CI [0.634, 0.657], cluster 6). While clusters with motifs of hydrophobic residues were expected to be enriched in strands, among aromatic residues, we observed no significant enrichment (cluster 5, highest AUROC = coil, 0.530 [0.517, 0.541]); among negative charged residues, disorder (cluster 7, AUROC = 0.610 [0.598, 0.622]); and among other hydrophobic, aromatic, or polar residues, strands (max. AUROC cluster 3, above).

### Motif position dependence

Treating the motif clusters as input for GIA, we performed an additional set of experiments to evaluate whether CANYA has learned positional information of motif effects and whether motif effects are additive (Fig. 7A). To learn positional information for each cluster of motifs, we ran an experiment in which we calculated the GIA effect of the cluster at every position of the construct, and compared it to the global, position-averaged effect of the cluster. These comparisons revealed that CANYA was also able to learn position-relevant information across each cluster of motifs (Fig. 7D). Regardless of the direction of their effects, motifs showed their strongest effect size within the first half of the construct and their weakest effect at the C terminus of the construct (Fig. 7D). The range of percent change was most drastic for clusters with a negative charge (cluster 6% change in effect from −79.52%, 95% CI [−80.94, −78.11] to 46.05% 95% CI [42.71, 49.31]; Fig. 7D) and weakest for clusters enriched in cysteines (−49.03% [49.66, −48.39] to 23.38% [21.84, 24.76] % change).

All positive effect clusters showed a dampened effect at the N terminus of the construct (minimum % change cluster 5 position 1 −24.33 [−27.31, −21.47]). Clusters 2, 3, 4, and 5 had their strongest increase of effect size at either position 7 or 8 (maximum change cluster 3 position 7 36.76% [34.33, 39.18]), whereas cluster 1 not only had a less substantial maximum change in effect size, but this change occurred in the center of the construct (position 10% change 23.39 [21.84, 24.76]).

Conversely, the negative-importance clusters generally had strengthened effects toward the N terminus, where the peptides are fused to Sup35N. Unlike other clusters, clusters 10 and 11 did not have their most dampened effect at the final position in the construct (position 18, note that the motifs are of length 3), but rather at position 17, perhaps because of their hydrophobic content. Cluster 11, which contains proline in the context of hydrophobic and polar residues, had the greatest increase in effect size closest to the N terminus (45.50% increase, 95% CI [41.70, 49.24]). Consistent with negative charges in the C terminus of some amyloid-forming peptides reducing fibril formation (*43*), cluster 7 showed a substantially less disruptive effect the closer it moved to the C terminus (minimum effect size position 18 −0.067 [−0.064, −0.07] compared to maximum effect size position 6 = −0.293 [−0.290, −0.300]). Cluster 9, the only cluster rich in tryptophan, showed very stable effects next to the N terminus before becoming its most aggregation-disruptive at position 9. We note that this insight was uniquely found using our interpretability methodology with CANYA, as examining amino acid enrichment differences between aggregators and non-aggregators would suggest increased effects of tryptophan closer to the N terminus (Fig. 2D).

All motifs followed a trend of dampened effects toward the C terminus of the construct. Many motifs also contained hydrophobic residues, and as enrichment of hydrophobicity toward the C terminus may affect the degradation rate of the sequence (*44*–*46*), we performed a follow-up experiment to determine whether these positional effects may have been biased simply because of different expression levels in sequences with hydrophobic C termini. In the experiment, we selected aggregators and non-aggregators with hydrophobic C termini and found that in selective conditions only requiring expression of Sup35N, both groups grew, suggesting that they are well expressed (fig. S10).

### Motif interactions

To next learn whether the effects of motifs were additive, we ran an experiment where we embedded motifs in a cluster in nonoverlapping positions between one and four times. Simple additive effects explained nearly all of the variance observed in model predictions (average *R*^2^ between multiplicity and importance = 0.99; Fig. 7B). However, some clusters showed evidence of heteroskedasticity (i.e., increased variance of effect as a function of increased motif counts) in their importance values, which may indicate minor epistatic or background-specific grammars.

Accordingly, we used GIA to perform an experiment similar to the one determining additivity of motifs; however, we focused on the case in which there are only two motifs, and the embedded motifs are selected from different clusters (Methods). This enables us to learn how interactions between clusters affect aggregation scores. Every cluster showed at least eight statistically significant interactions [*P* value of paired, two-way *t* test <0.05/(11*11 tests); Fig. 7C; Methods], suggesting the importance of modeling sequence context in the prediction of aggregation status. Nonetheless, cluster interaction effects were modest (ranging from −0.051 to 0.067) compared to cluster main effects (−0.140 to 1.06). All clusters exhibited a self-enhancing effect in which their interaction importance was significantly higher than the importance from additively combining each marginal effect (maximum importance cluster 2, 0.067, minimum cluster 9 0.003). Positive effect motifs interacted with positive motifs to increase aggregation propensity beyond expected from marginal presence of either motif, and negative effect motifs interacted with other negative effect motifs reducing the expected disrupting effect from marginal presence of both motifs (i.e., with both motifs, the sequence is more likely to aggregate than expected). Contrastingly to positive effect motifs, this perhaps suggests that negative effect motifs follow a diminishing returns-type phenomenon. Nonetheless, clusters 3 and 11 were the exception of two motifs of opposite signs interacting positively (importance 0.0035, *P* value <2 × 10^−16^). Despite similar physicochemical properties, clusters 2 and 3 displayed different interaction effects. Cluster 3, with a preference for aromatic over hydrophobic residues, had its strongest interaction with cluster 4 (0.064, compared to cluster 2-cluster 4 0.039), while the top interaction for cluster 2 was with itself. The strongest negative interaction occurred between the cysteine-rich cluster 1 and cluster 8, containing positive and negative charged residues among polar residues (importance −0.051, *P* value <2 × 10^−16^).

## DISCUSSION

Amyloid protein aggregation is a hallmark of many human diseases and a major problem in biotechnology. However, relatively few protein sequences are known to aggregate under physiological conditions, and this shortage of data likely limits our ability to understand, predict, engineer, and prevent the formation of amyloid fibrils.

Here, we have directly addressed this data shortage by quantifying aggregation at an unprecedented scale (100,000 random sequences) and used the data to evaluate the performance of existing computational models. Finding the performance of these methods to be limited, we then used the data to train CANYA, a fast and interpretable deep learning model of amyloid aggregation. Evaluation on an additional independent 7000 sequences confirmed the performance of CANYA on predicting aggregation from sequence.

Using random sequences allowed us to test the aggregation of sequences very different to the small number of known amyloids and to provide a principled evaluation of existing amyloid predictors (*12*–*15*, *23*) over both our own and existing datasets (*23*, *31*, *36*, *47*), serving as a guideline for the community. The performance of CANYA and its consistency across evaluation tasks suggests that CANYA does indeed learn an accurate approximation of the sequence-aggregation landscape, despite only training on random, synthetic peptides. The performance of previous methods compared to that of hydrophobicity scales suggests that the use of limited dataset sizes and short peptides has limited the amount of additional aggregation-relevant information these approaches could learn. Perhaps a composition of only a few amino acids (e.g., hexapeptides) is too short to learn position-specific or motif-interactive effects, as there is little to no additional context on which to model these effects. Hence, hydrophobicity explains the majority of variance of aggregation rates in these shorter sequences. This underscores the importance of using longer sequences and high-throughput assays to profile previously unexplored regions of the sequence-aggregation landscape.

CANYA has an inherently interpretable model whose architecture is inspired by biology. We also adapted state-of-the-art xAI techniques from genomic neural networks to the protein space (*33*, *34*, *37*, *48*). This not only reveals insights into the decision-making process of our model but also illustrates how xAI techniques developed for genomic neural networks can provide intelligible information from neural networks that model protein function.

Interpretability analyses identified “physicochemical motifs” that underlie CANYA’s decision-making process, including aggregation-promoting motifs enriched in β strands of known amyloid structures and aggregation-preventing motifs enriched in disordered regions of known amyloids. The effects of these physicochemical motifs combined mostly additively, with only subtle motif-motif interactions, suggesting a modest role for long-range epistasis or context specificity in the process of amyloid aggregation. However, the physicochemical motifs did have position-specific effects, and these warrant additional investigation in future experimental work.

A potential limitation of our strategy is that we only tested the aggregation of sequences of 20 amino acids and in one particular context. Primarily, our learned motifs and their effects are approximations learned from the context of our experimental assay, in which Sup35 is appended at the N terminus. There likely remains additional predictive power to be harvested by experimentally testing at scale, as well as modeling longer sequences and consequently longer-range interactions. Moreover, we note that Sup35N is a disordered protein, likely leaving the 20-residue construct exposed. In contrast, amyloidogenic sequences are often buried in the interior of folded proteins and aggregation additionally requires destabilization of the native state. Nonetheless, we found through several evaluations that the information learned by modeling the length-20 constructs from our experimental assay can accurately predict aggregation-prone regions across a variety of protein lengths and contexts.

An additional consideration is CANYA’s architecture. We limited our neural network architecture to a relatively simple class of models as our focus was on interpretability. Recent literature suggests that leveraging protein embeddings—in lieu of one-hot encoding sequences—may boost our predictive power (*49*–*56*), although such an approach will likely pose difficulties when performing post hoc xAI experiments as done here (*57*). Further, our model comprises a modest 17,000 parameters and leverages sparsity despite having more than 100,000 sequences on which to learn. Many models of protein structure use much more complex architectures, with both substantially larger numbers of layers and parameters (*49*, *54*, *56*, *58*–*60*). Future investigations may build off of the work presented here by generating longer sequences or exploring more complex architectures.

The pairing of massive-scale experimental data generation using random sequences with interpretable models has led to insights into genomic regulatory functions (*61*). However, to the best of our knowledge, it has been little used in the space of proteins to probe mechanisms beyond short motifs. We believe that the approach deserves wider adoption, whenever sequences are functional at sufficient frequencies to allow their identification in practical library sizes. For example, in future work, it will be interesting to quantify at scale how the sequences of peptides alter their cellular concentration and to what extent this contributes to other molecular phenotypes such as aggregation propensity. Systematic large datasets such as the one presented here can be reused to train and evaluate additional models, and the predictions and outputs of these models can loop back into additional large-scale experimental explorations of sequence space.

## METHODS

### Plasmid library construction

Libraries of random sequences (NNK1–4) were synthesized by Integrated DNA Technologies (IDT) as ultramers of 20 NNK codons (60 nucleotides, nt). A library containing 400 sequences selected from the previous four random libraries was synthesized as an oligopool by IDT for validation and replication (data files S3 and S4). In both cases, sequences were flanked by constant regions of 25 nt upstream and 21 nt downstream for cloning. The NNK ultramers and the replication oligo pool were extended in a one-cycle polymerase chain reaction (PCR) (Q5 high-fidelity DNA polymerase, NEB) with primers TSO_2 and TSO_65 (data file S5). The resulting products were treated with 2 μl per tube of ExoSAP (ExoSAP-IT, Applied Biosystems) for 30 min at 37°C and 20 min at 80°C and purified through a MinElute column (Qiagen). In parallel, the PCUP1-Sup35N plasmid was linearized by PCR (Q5 high-fidelity DNA polymerase, NEB; primers TSO_3 and TSO_4; data file S5). The products were purified from a 1% agarose gel (QIAquick Gel Extraction Kit, Qiagen) and ligated by Gibson with 3 hours of incubation at 50°C followed by dialysis for 3 hours on a membrane filter (MF-Millipore 0.025 μm membrane, Merck) and vacuum concentration. The resulting (NNK1–4) libraries were transformed into 10-beta Electrocompetent *Escherichia coli* (NEB), by electroporation with 2.0 kV, 200 ohm, 25 μF (Bio-Rad GenePulser machine). The cells were recovered in super optimal broth with catabolite repression (SOC) medium for 30 min and grown overnight in 50 ml of LB ampicillin medium. A small amount of cells was also plated on LB ampicillin plates to assess transformation efficiency. Total transformants were estimated (data file S6), and 50 ml of overnight culture were harvested to purify each library with a midi prep (Plasmid MIDI Kit, Qiagen). Libraries NNK1 to 4 were bottlenecked to ~1 million transformants, while for the replication library, we estimated 625,000 transformants.

### Large-scale yeast transformation of random libraries

*Saccharomyces cerevisiae* GT409 [psi-pin-] (MATα ade1–14 his3 leu2–3,112 lys2 trp1 ura3–52) provided by the Chernoff laboratory was used in all experiments in this study (*28*). Yeast cells were transformed with the above plasmid library midi preps. After an overnight pregrowth culture in 25 ml of yeast peptone dextrose adenine (YPDA) medium at 30°C, the cells were diluted to optical density at 600 nm (OD_600_) = 0.3 in 175 ml of YPDA and incubated at 30°C 200 rpm for ~4 hours. When the cells reached the exponential phase, they were harvested, washed with Milli-Q, and resuspended in sorbitol mixture [100 mM LiOAc, 10 mM tris (pH 8), 1 mM EDTA, and 1 M sorbitol]. After a 30-min incubation at room temperature (RT), 4 μg of plasmid library and 175 μl of ssDNA (UltraPure, Thermo Fisher Scientific) were added to the cells. Polyethylene glycol (PEG) mixture [100 mM LiOAc, 10 mM tris (pH 8), 1 mM EDTA (pH 8), and 40% PEG3350] was also added and cells were incubated for 30 min at RT and heat-shocked for 15 min at 42°C in a water bath. The cells were harvested, washed, resuspended in 250 ml of recovery medium [YPD, sorbitol 0.5 M, and adenine (70 mg/liter)], and incubated for 1.5 hours at 30°C 200 rpm. After recovery, the cells were resuspended in 350 ml of synthetic complete medium lacking uracil (SC-URA) and allowed to grow for 50 hours. Transformation efficiency was calculated for each of the four transformations by plating an aliquot of cells in SC-URA plates (data file S6). Two days after transformation, the culture was diluted to OD_600_ = 0.08 in 500 ml SC-URA medium and grown until exponential phase. At this stage, the cells were harvested and stored at −80°C in 25% glycerol. In yeast, libraries NNK1–4 were bottlenecked to 0.5 to 1 million transformants (data file S6).

### Small-scale yeast transformation of replication library

Yeast cells were transformed with the library containing 400 sequences in three biological replicates. An individual colony was grown overnight in 3 ml of YPDA medium at 30°C and 4*g*. Cells were diluted in 60 ml to OD_600_ = 0.25 and grown for 4 to 5 hours. When cells reached the exponential phase (OD ~ 0.7 to 0.8), the cells were harvested at 400*g* for 5 min, washed with Milli-Q, and resuspended in 1 ml of YTB [100 mM LiOAc, 10 mM tris (pH 8.0), and 1 mM EDTA]. They were harvested again and resuspended in 72 μl of YTB. One hundred nanograms of plasmid library was added to the cells, together with 8 μl of salmon sperm DNA (UltraPure, Thermo Fisher Scientific) previously boiled, 60 μl of dimethyl sulfoxide (Merck), and 500 μl of YTB-PEG [100 mM LiOAc, 10 mM tris (pH 8.0), 1 mM EDTA, and 40% PEG 3350]. The cells were incubated at RT for 30 min at 4*g*. Heat shock was performed at 42°C for 14 min in a thermo block. Last, the cells were harvested and resuspended in 50 ml of SC-URA medium, allowing them to grow for 50 hours at 30°C and 4*g*. A small amount of cells was also plated in plasmid selection medium to assess transformation efficiency. We estimated 70,000 transformants per replicate (data file S6). Two days after transformation, the culture was diluted to OD_600_ = 0.08 in 500 ml of SC−URA medium and grown until exponential phase. At this stage, the cells were harvested and stored at −80°C in 25% glycerol.

### Selection experiments

Cells were thawed from −80°C in 50 ml of plasmid selection medium at OD = 0.05 and grown until exponential for 15 hours. At this stage, the cells were harvested and resuspended in 300 ml of protein induction medium (SC-URA, 2% glucose, and 100 μM Cu_2_SO_4_) at OD = 0.1. After 24 hours, 250 ml of input pellets were collected, and cells were plated on synthetic complete medium lacking uracil and adenine(SC-ADE-URA) in 145-cm^2^ plates (Nunc, Thermo Fisher Scientific). The plates were incubated at 30°C for 7 days. Last, the colonies were scraped off the plates with phosphate-buffered saline 1× and harvested by centrifugation to collect the output pellets. Both input and output pellets were stored at −20°C before DNA extraction. For each random library experiment, one input sample and three technical replicates of the output pellet were processed for sequencing. Selection experiments for the replication library were instead performed in three biological replicates, following the same steps as above. Three input and three output samples were processed for sequencing.

### Spot dilution assays

GT409 [psi-pin-] and GT159 [psi-PIN+] (*28*) were transformed with plasmids expressing SupN fused to eight sequences with different aggregation propensity. For measuring growth in selective conditions, yeast cells expressing individual variants were grown overnight in plasmid selection medium (SC-URA 2% glucose). They were then diluted to OD 0.1 in protein induction medium (SC-URA 2% glucose 100 μM Cu_2_SO_4_) and grown for 24 hours. Twenty millions cells (OD ~ 1) were used as the starting concentration (dilution 1:1), and serial dilutions 1:10 were carried out to reach a 1:10,000 dilution. Three microliters of each dilution was plated on SC-URA (control) and SC-ADE-URA (selection) plates and allowed to grow for 7 days at 30°C before pictures were taken with a GelDoc XR (Bio-Rad).

### DNA extraction and sequencing library preparation

Input and output pellets were thawed and resuspended in 1.5 ml of extraction buffer [2% Triton X-100, 1% SDS, 100 mM NaCl, 10 mM tris (pH 8), and 1 mM EDTA (pH 8)] and underwent two cycles of freezing and thawing in an ethanol-dry ice bath (10 min) and at 62°C (10 min). Samples were then vortexed together with 1.5 ml of phenol:chloroform:isoamyl 25:24:1 and 1.5 g of glass beads (Sigma-Aldrich). The aqueous phase was recovered by centrifugation and mixed again with 1.5 ml of phenol:chloroform:isoamyl 25:24:1. DNA precipitation was performed by adding 1:10 V of 3 M NaOAc and 2.2 V of 100% cold ethanol to the aqueous phase and incubating the samples at −20°C for 1 hour. After a centrifugation step, pellets were dried overnight at RT. The pellets were resuspended in 900 μl of resuspension buffer [10 mM tris (pH 8) and 1 mM EDTA (pH 8)] and treated with 7.5 ml ribonuclease A (Thermo Fisher Scientific) for 30 min at 37°C. The DNA was finally purified using 30 μl of silica beads (QIAEX II Gel Extraction Kit, Qiagen), washed, and eluted in 22 μl of elution buffer. Plasmid concentrations were measured by quantitative PCR with SYBR green (Merck) and primers annealing to the origin of replication site of the PCUP1-Sup35N plasmid at 58°C for 40 cycles (TSO_05 and TSO_06; data file S5). The library for high-throughput sequencing was prepared in a two-step PCR (Q5 high-fidelity DNA polymerase, NEB). In PCR1, 160 million plasmid molecules were amplified for 15 cycles at 68°C with frame-shifted primers with homology to Illumina sequencing primers (primers TSO_7 to TSO_20; data file S5). The products were purified with ExoSAP treatment (Affymetrix) and by column purification (MinElute PCR Purification Kit, Qiagen). They were then amplified for 10 cycles in PCR2 with Illumina-indexed primers (primers TSO_21 to TSO_54; data file S5). The library was sequenced by 150–base pair paired-end sequencing in an Illumina NextSeq500 sequencer at the CRG Genomics core facility. See data file S7 for read counts and distributions across NNK experiments.

### Sequence data preprocessing

We processed each of the four NNK experiments separately using DiMSum (*62*). Briefly, DiMSum comprises an end-to-end pipeline for processing deep mutational scanning datasets from raw reads to measured sequences and their associated assay scores (plus errors). DiMSum was run with the following parameters: cutadaptMinLength = “60”; cutadaptErrorRate = “0.2”; vsearchMinQual = “30”; vsearchMaxee = “0.5”; startStage = “0”; fitnessMinInputCountAny = “0”; maxSubstitutions = “20”; mixedSubstitutions = “TRUE”; experimentDesignPairDuplicates = “TRUE.” We then removed sequences with fewer than 100 reads in the input sequencing experiment. Next, we centered the fitness estimates (aggregation scores) of each dataset individually by adding or subtracting the corresponding mode fitness of the non-aggregating sequences. After centering each sequence, we next labeled sequences as “aggregators” (or “non-aggregators”) by transforming their fitness estimate to a *z* score composed of the fitness estimate scaled by the DiMSum error and performing a one-sided hypothesis test to check whether the standardized score was significantly larger than 0. We treated sequences whose *P* values after FDR adjustment were ≤ 0.05 as aggregators and the remaining sequences as non-aggregators. A proportion of sequences with >100 input reads produced no reads after the selection experiments, thus leading to NA scores from DiMSum. We labeled these sequences as non-aggregators. If a sequence contained a stop codon, we used only the component of the sequence preceding the stop for model training. For cases in which this resulted in duplicate sequences (e.g., FN*VILRDEGHGSYGFDNNN and FN*FVVMHTCIMVVFCLGDI are both mapped to “FN”), we summarized the truncated sequence by taking its mean aggregation score or mode aggregation status across observations. If a given truncated sequence had an equal number of aggregator and non-aggregator status observations, we discarded this truncated sequence. As a result, we classified >35,000 sequences for libraries NNK1 to 3 (35,456; 37,578; 38,893 respectively) and 7040 for NNK4.

### The architecture of CANYA

CANYA is a biologically motivated hybrid-neural network designed to find motifs and their interactions. More concretely, the architecture of CANYA is inspired by recent work that suggests that stacked convolution and attention layers serve as a reasonable inductive bias for motif and motif-interaction discovery. The hyperparameters of CANYA were influenced by summary statistics of interacting secondary structure elements in amyloids within the PDB (fig. S5). Summarily, we chose the simplest architecture of our model such that it is expressive, interpretable, and principled in biological knowledge.

CANYA takes as input an amino acid sequence of length limit up to 20 residues, and outputs a score related to the sequence’s propensity to form amyloids. Before passing the sequence to the input layer, we first one-hot encode it, allowing only the 20 canonical amino acids. As we use filters of length 3 (see below for justification; fig. S5), we pad the sequence with two 0s both up- and downstream the sequence. Finally, if this padded sequence is not of length 24, we add a mask with values of −1 downstream the sequence until it reaches length 24. The input length restrictions of CANYA arise from the fact that a given sequence in the assay is (up to) length 20 and is padded with two 0 s on each side. Explicitly, the training data of CANYA look as follows

00[one-hot encoded random sequence]00

when there is no masking or stop codons, and as follows if so

00[one-hot encoded random sequence]00 [-1]

where the number of −1 values is the required quantity such that the sequence is length 24.

The input layer of CANYA correspondingly accepts a matrix of size 24 × 20 representing a one-hot encoded, padded, and potentially masked peptide sequence. The output layer is a single unit with sigmoid activation. The hidden layers of CANYA are:

1) Convolution (100 filters, size 3, stride 1, exponential activation).

2) Self-attention (1 attention head, key-length 6).

3) Fully connected layer [64 units, Rectified Linear Unit (ReLU) activation].

We selected an exponential activation function for the convolutional layer as this type of activation is generally more robust for motif discovery (*63*). We chose filters of length 3 as this was the mode length of β sheets in amyloid sequences with resolved structures in UniProt (fig. S5). We use dropout with probability 0.1 after the convolution and attention layers and 0.4 after the fully connected layer. We use an elastic net regularization (with value 0.01) when learning the weights between the attention and fully connected layers. Lastly, to encourage the model to learn positional information, we do not perform pooling after the convolution layer, and we include positional encodings before taking the softmax in the attention layer. We trained CANYA for 100 epochs using the Adam optimizer with default values and the binary Kullbeck-Leibler (KL) divergence as a loss function. We limited the learning rate of the model during training by monitoring the validation area under precision-recall curve, decaying at a factor of 0.2 with patience 4, and performed early stopping by monitoring the validation area under precision-recall curve with patience 10. For sequences with length greater than 20, we collect the CANYA score at every overlapping length-20 window of the sequence and then use its median CANYA score as its final score. We note that other methods may report the minimum score, or 0, (perhaps under the logic that aggregation-forming propensity is limited by a sequence’s most aggregation-disrupting region). Consequently, we performed an experiment where we varied this summarizing function (including the maximum, mean, and minimum) and show that this function may affect performance (fig. S11). Nonetheless, the relationship of length and aggregation-forming propensity is nontrivial, for which we suggest using the median as the summarizing function, owing to its stability.

### Compilation of external datasets

We first collected 6-mers from the WALTZ-DB dataset (*31*). Here, we assigned all sequences whose “Classification” field was “amyloid” as a 1, and all other sequences as 0. We next collected the aggregating peptides from the CPAD repository (*36*). We used sequences from the “Peptide” field, filtering for sequences of at least length 10 and for sequences that did not contain a space in their sequences. We assigned sequences with “Classification” field amyloid a 1, and all other sequences 0. The final external dataset we used was from the AmyPro database (*47*). All sequences in the AmyPro dataset were amyloids, and so we sought to evaluate methods’ abilities to distinguish the amyloidogenic region from the non-amyloidogenic regions of the sequences. First, we collected all sequences from the “regions” field in the dataset. Next, we removed each of these “region” sequences from the main peptide sequence and concatenated the remaining two portions of the main sequence together, comprising a set of positive sequences (labeled 1) from the “regions” field and negative sequences (labeled 0) from the remaining peptide sequences. Finally, we constructed our negative sequences by breaking the concatenated sequences into subsequences of length equal to their corresponding amyloidogenic (positive) sequences. This led to a substantial case-control imbalance, for which we sampled from the non-amyloidogenic sequences a number of negative samples equal to the number of positive samples. While this task evaluates unnatural sequences, it evaluates the ability of each method to distinguish amyloid cores from non-amyloid cores. We list descriptive summary statistics (e.g., length, sample sizes, and hydrophobicity) in table S1.

### Aggregation predictors

Aggregation predictors or physicochemical scales [Tango (*12*), Amypred (*23*), Camsol (*13*), PLAAC (*14*), and Aggrescan (*15*)] were used to calculate a score for each sequence. When appropriate, individual residue-level scores were summed to obtain a single score per sequence. CamSol, Amypred, and Aggrescan were run with the default parameters. PLAAC was run using a core of length 6 and weightings from input sequences. Tango was run with pH 7.2, no protection of termini, ionic strength = 0.1, and T = 298 K (25°C). Some of the predictors present sequence-length limitations: Amypred runs only for sequences longer than 10 amino acids, CamSol for sequences longer than 6 amino acids, and Aggrescan cannot be run for sequences longer than 2004 amino acids. We note that several of these methods (including TANGO, CamSol, and Aggrescan) were directly trained on sequences within CPAD and other datasets presented in the manuscript, violating the ability to evaluate their out-of-sample predictive performance on these datasets. This complication is exacerbated by several methods (e.g., TANGO and CamSol) also being ensemble methods (or extensions) that leverage several algorithms for prediction—it is not trivial to account for, or remove, these previously seen sequences, as any sequence that was used for training the main algorithm or their antecedent ensemble methods is not out-of-sample.

### Selecting a model for interpretability analyses

We trained CANYA with random weight initialization 100 times and recorded for each fitted model the area under the receiver-operating characteristic curve (AUROC) of the test data, area under the precision-recall AUPR of the test data, and interpretability score adapted from a recently developed approach for interpretability analyses of genomic neural networks (*34*). Briefly, Majdandzic *et al.* propose an approach to quantify the consistency of the attribution maps of a trained model by comparing the entire set of kmers in the training sequences to the set of kmers in [adjusted (*48*)] attributed positions in the training sequences. These two distributions of kmers—in the case of CANYA, 3-mers—are compared using the KL divergence, where a higher KL divergence suggests greater amenability to downstream interpretability analyses. To calculate an interpretability score for each trained instance of CANYA, we used this same approach, but rather than using kmers of nucleotides, we used kmers from the input amino acids. As we saw that the test AUPR was more consistent across experiments, we used a models’ mean AUPR across experiments and interpretability score as model selection criteria. More rigorously, we selected the model with the highest interpretability score, conditional on the fact that its mean AUPR across datasets was greater than the median of these mean scores across model training instances.

### Visualization of filters (motifs)

Notably, the use of random sequences in amino acid space poses difficulties for observing a typical, lexicographic motif, and consequently, observing convergence toward a lexicographic motif in first-layer convolutional filters. We elaborate as follows: Using a filter length of 3, there is a 1 in 8000 (20^3^) chance of observing a given kmer. Ideally, for the model to learn a stable feature, this kmer must not only exist in a sizable proportion of sequences, but its effect must also not be masked out by surrounding contextual information. Even if we were to ignore contextual information, this motif would need to occur independently multiple times, an event whose probability quickly converges to 0. Consequently, we are much stricter than previous approaches when generating a PWM for a given filter. For interpretability’s sake, we limit the kmers comprising a PWM for a filter to the minimum of either the 10 most-activating kmers of a filter, or the collection of kmers whose activation is at least 75% of the maximum-activating kmer. Summarily, a filter is both visualized and represented numerically by its PWM composed of at most the top 10 strongest activating kmers.

### Motif clustering

Following the above logic, CANYA must learn physicochemical properties of amino acids and understand how these properties interact among each other when constructing its features at the convolution layer. Moreover, these physicochemical 3-mers, or motifs, may often capture redundant physicochemical information, but independent sequences—for example, two different motifs capturing hydrophobicity, may separately comprise sequences of “IVF” or “ALM.” To further improve interpretability and to reduce the dimensionality of downstream experiments leveraging the learned motifs of CANYA, we performed clustering on the PWM matrices. More concretely, we calculated BLOSUM scores for each filter by taking the dot product between its PWM and BLOSUM score matrix (*41*). We next performed affinity propagation on these calculated motif BLOSUM scores to cluster the motifs. Affinity propagation found 11 clusters of motifs. However, after performing GIA experiments (*37*), we found seven discrepancies when evaluating whether a given motif had the same effect size (importance score) direction compared to the effect size of the motif with the greatest absolute effect within the cluster. As our goal was to interpret model decisions and physicochemical clusters, we removed these seven filters from their corresponding clusters so that each cluster contained only filters with the same effect size direction. We show the original and changed cluster assignments in fig. S7.

### GIA experiments

To learn the effect of motif presence on CANYA’s decision-making, we turned to GIA in silico experiments (*37*). Briefly, GIA is a post hoc interpretability method applied to genomic neural networks that enables users to learn importance scores (i.e., effect sizes) of a given sequence feature on a model’s output score. The importance score is derived from taking the average difference in model score between a set of background sequences, and this same set of background sequences but with a functional element, such as a motif, placed in the background sequence (sequence length is maintained, i.e., a window of the sequence is replaced by the functional element). For all experiments, we limited our analyses to 25,000 randomly selected, full-length (length-20 and absent of stop codons) training sequences that were confidently predicted by CANYA. We defined “confidently predicted” as aggregators with CANYA score above 0.3 and non-aggregators with CANYA score below 0.2 (see fig. S12 for prediction score distributions). Finally, we emphasize that owing to the random nature of our experiment, the training sequences serve as a valid set of background sequences for GIA as they span an extremely wide range of contexts.

In the first set of GIA experiments, we sought to characterize the importance score of each filter individually. To do so, we first randomly selected 25,000 sequences from the training set, comprising sequences from across all three experiments. Next, for a given filter, we collected the activation energy of each kmer used to represent the PWM and used the ratio of the activation energy of each kmer to the activation energy of the kmer with the maximum activation energy in this PWM to generate kmer sampling probabilities. For each sequence, we randomly sampled one kmer using this normalized ratio as the kmer’s sampling probability and embedded this kmer into the sequence. Afterward, we calculated for all 25,000 background sequences and all 25,000 modified sequences the CANYA aggregation score before applying the softmax function. We calculated each filter’s importance score as the mean paired difference in scores between the 25,000 background and modified sequences.

After clustering the learned motifs, we next wished to validate whether the clusters could be used to simplify further interpretability analyses by reducing the scale of in silico experiments performed. To do so, we conducted a GIA experiment within each cluster to determine a cluster-level importance score. The experiment follows the same logic as the original, filter-level GIA experiment, only that we first randomly selected a filter within a cluster before sampling a kmer from its PWM. The filters were randomly selected according to the ratio of their absolute GIA importance score to the maximum absolute GIA importance score across filters of the corresponding cluster. Indeed, cluster-level scores recapitulated the scores of the motifs from which they were composed (table S6). We therefore performed all following GIA analysis at the cluster level, using this filter-first, kmer-second sampling scheme.

We next performed an experiment to evaluate the additivity of motif clusters on aggregation propensity. Here, we collected 25,000 background sequences from the training dataset and then embedded into these background sequences 1 to 4 kmers in nonoverlapping positions where each of the 4 kmers was sampled using the filter-first, kmer-second sampling scheme. Each sequential kmer addition (from kmers 2 to 4) was embedded in the sequence such that the sequence with antecedent kmer multiplicity maintained the kmer(s) at its (their) original embedded position(s). We calculated the cluster importance score for a given multiplicity by taking the mean difference in prediction score between the sequences with the injected kmer(s) and their corresponding background sequences—in other words, each importance score is generated by taking the mean difference between 25,000 background sequences and 25,000 modified background sequences with either 1, 2, 3, or 4 embedded kmers.

To evaluate whether CANYA learned position-specific importance of motifs, we performed an additional GIA experiment in which we systematically embedded a motif cluster at each position of a random sequence. In these experiments, we performed a single GIA experiment with 25,000 background sequences and 25,000 modified sequences for each position from positions 1 to 18 so that the entire 3-mer could be contained within the sequence.

In a final GIA experiment, we characterized interaction effects between motif clusters. For a given motif cluster pair, we sampled a kmer (as mentioned above) from each cluster as well as a corresponding position randomly from positions 1 to 18 in which to embed each kmer. We evaluated the CANYA score for the background sequence, the background sequence with the kmer from the first cluster at the first sampled position, the background sequence with the kmer from the second cluster at the second sampled position, and the background sequence with both kmers at both positions. We called the interaction importance as the result of subtracting the sum of CANYA predictions of the sequences with each marginal kmer embedding from the sum of the CANYA predictions of the background sequence and sequence with both motifs. The final importance was calculated as the mean interaction importance across 25,000 sequences.

### Secondary structure enrichment scoring of motifs

To examine whether certain motifs were characteristically similar to sequences found in specific secondary elements of amyloids, we examined activation energies of filters across secondary structure elements in a set of amyloids with resolved structures in the PDB. Concretely, we collected 114 entries from the StAmP dataset (*42*) and then downloaded their structural information from the PDB (see table S7 for entries and corresponding proteins). Next, we passed all sequences through CANYA and extracted their filter activation energies (i.e., output from the convolution layer). At each position, we summarized a cluster’s activation energies as the maximum activation energy across filters within a cluster, generating a vector of maximum activation energies for each cluster. Next, we encoded each secondary structure (coil, β strand, or disorder) as a binary vector where 1 indicated positions in the corresponding secondary structure, and 0 indicated otherwise. We collected this set of secondary structure vectors and activation energy vectors for all sequences and then concatenated them across sequences. Last, we generated secondary structure enrichment scores by calculating the AUROC between a given secondary structure element and cluster activation energy across all sequences.
