# Supplementary material for: Massive experimental quantification allows interpretable deep learning of protein aggregation
Source: Sci Adv. 2025 Apr 30;11(18):eadt5111. doi: 10.1126/sciadv.adt5111 (PMC12042874; doi:10.1126/sciadv.adt5111)
Supplement: Supplementary file 1 — Figs. S1 to S12 Legends for data files S1 to S7 Legends for tables S1 to S7 [file sciadv.adt5111_sm.pdf]

Science Advances
AAAS

Supplementary Materials for

Massive experimental quantification allows interpretable deep learning of protein aggregation

Mike Thompson *et al.*

Corresponding author: Benedetta Bolognesi, bbolognesi@ibecbarcelona.eu; Ben Lehner, bl11@sanger.ac.uk

Sci. Adv. **11**, eadt5111 (2025)
DOI: 10.1126/sciadv.adt5111

The PDF file includes:

Figs. S1 to S12
Legends for data files S1 to S7
Legends for tables S1 to S7

Other Supplementary Material for this manuscript includes the following:

Data files S1 to S7
Tables S1 to S7

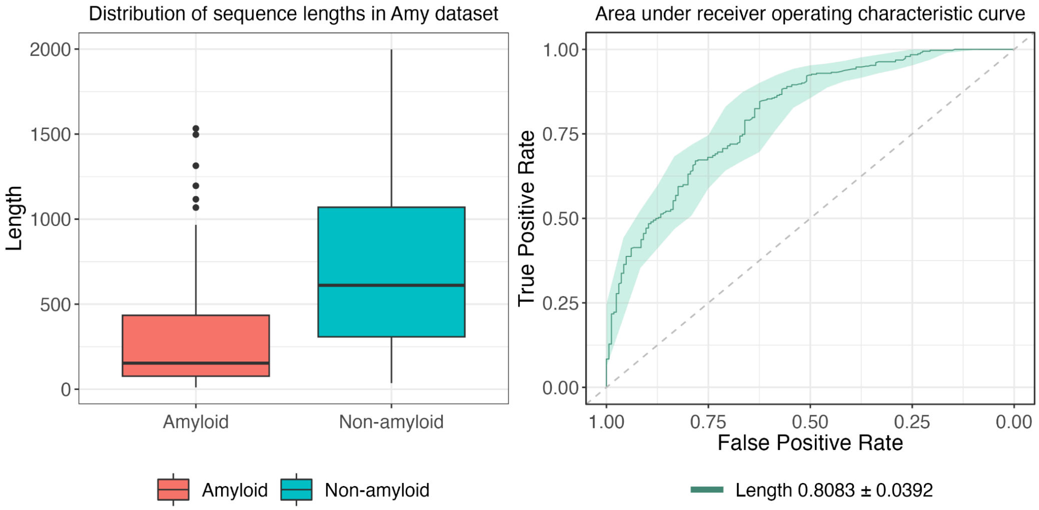

Supplementary Figure 1 Sequence length trivially predicts the Amy dataset. Several previous tools for predicting amyloid-forming propensity have been trained on the Amy dataset. However, the dataset presents grave concern for model training and evaluation due to the bias in sequence-length distribution between classes.

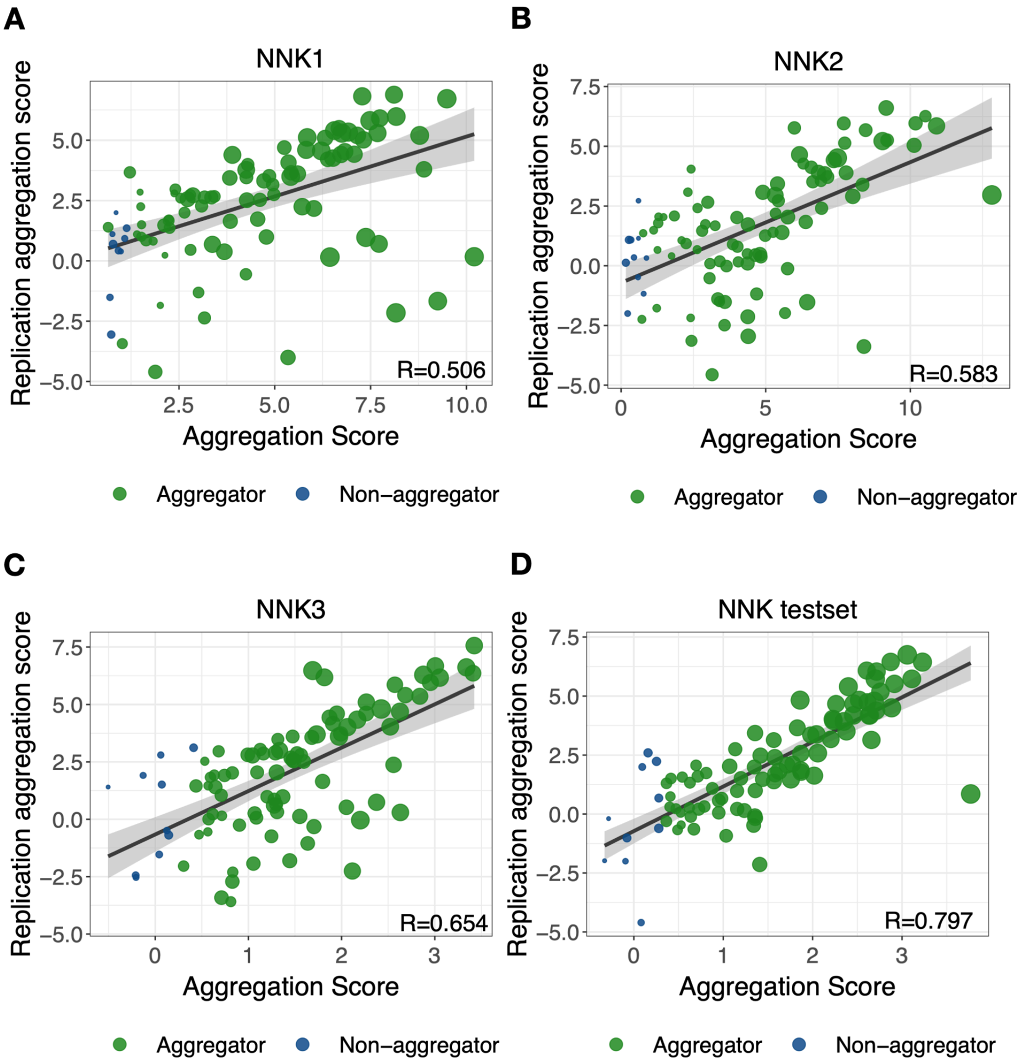

Supplementary Figure 2 Aggregation scores are replicable across experiments. Aggregation scores from the corresponding original experiment are plotted on the x-axis, and the replication aggregation score is plotted on the y-axis. Sizes are proportional to the inverse error measurement from the original experiment as reported by DiMSum. The x-axis scores were all calculated independently within their respective experiment—(A) NNK1, (B) NNK2, (C) NNK3, (D) NNK4, the validation set—and altogether in the replication set (n=100), as 100 sequences were taken from each experiment. We note that the replication experiment contained several orders of magnitude fewer sequences than the original experiments. As our assay is competition-based, the distribution of fitness scores may be non-trivially transformed (compared to the original distributions in which the vast majority of sequences did not aggregate). However the rank is preserved across replicates, as is the variance explained by the replication.

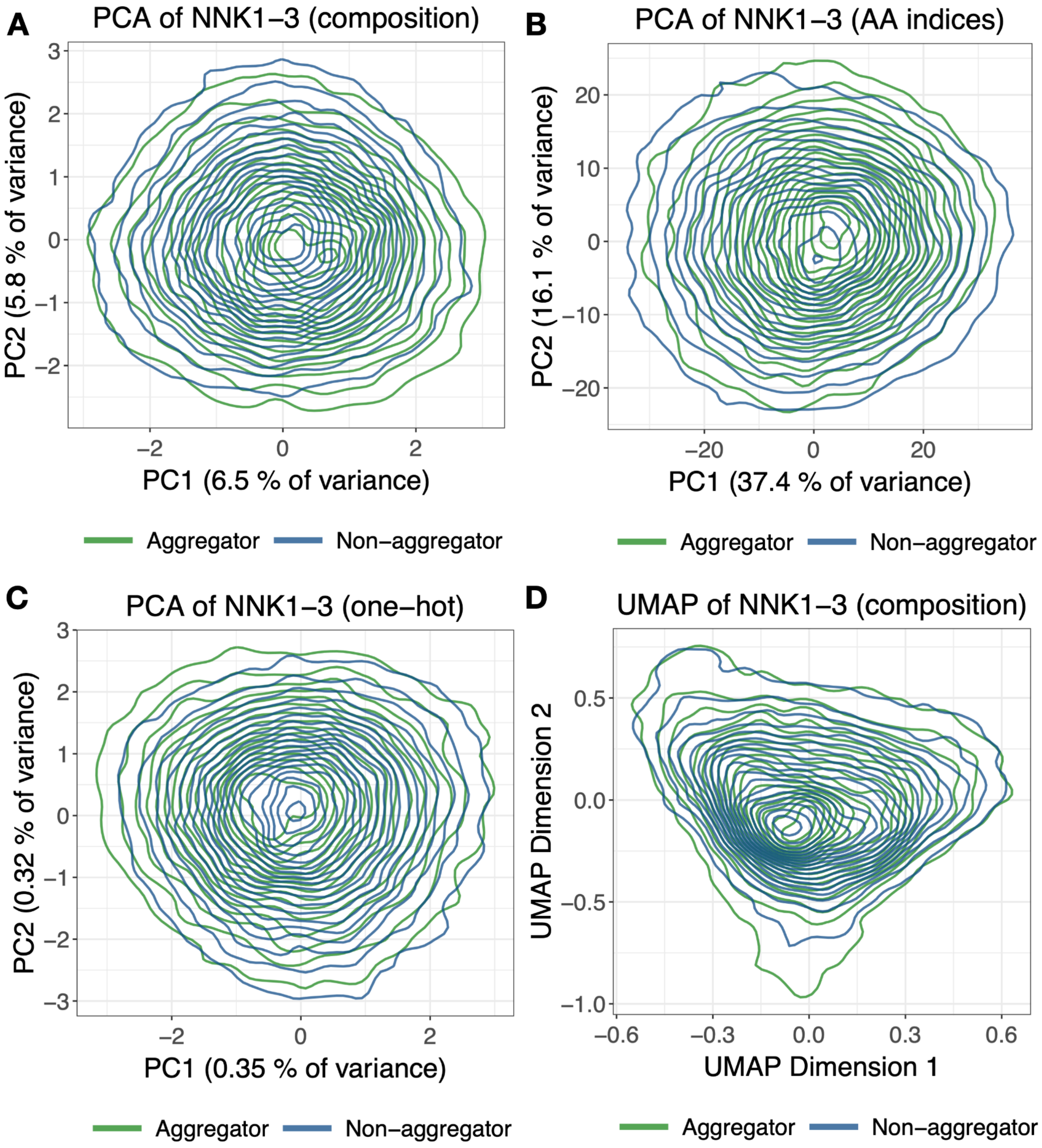

Supplementary Figure 3 Dimensionality techniques fail to distinguish aggregation status. (A-C) Contour plots of Principal Component Analysis (PCA) scores on PCs 1 and 2 when sequences are represented by (A) overall amino acid composition, (B) 533 amino acid indices calculated from python package protlearn (C) one-hot (position-maintained) amino acid composition. (D) UMAP projection when using amino acid composition as input.

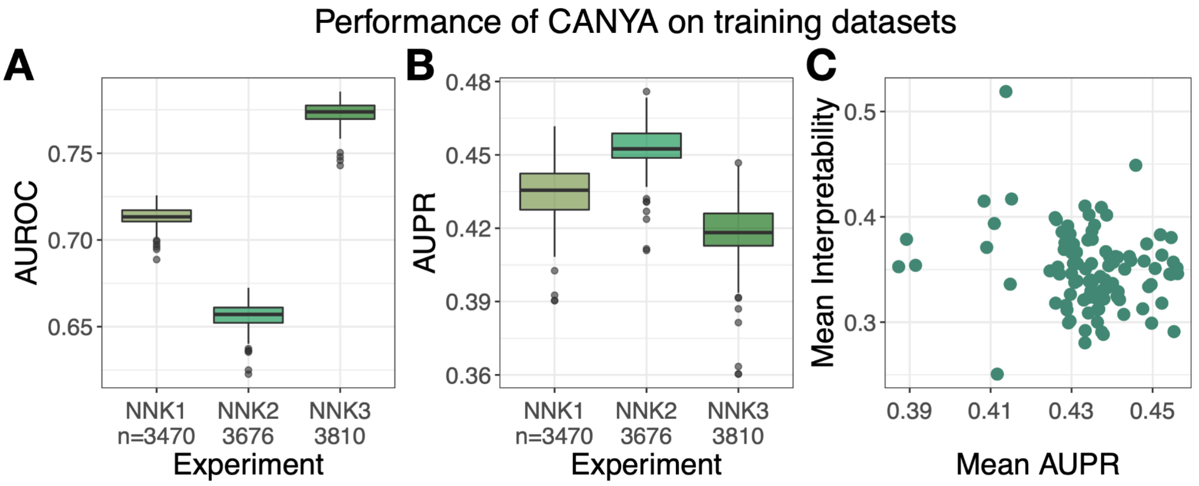

Supplementary Figure 4 CANYA performance on the held-out test portion of the training datasets. Evaluation metrics across the all 100 model fits of CANYA. (B) The area under receiver operating characteristic curve (AUROC) for held-out testing sequences. (C) The area under precision recall curve (AUPR) for held-out testing sequences. (D) The interpretability score (KL divergence; Methods) calculated on all held-out test sequences plotted against the mean AUPR across training experiments.

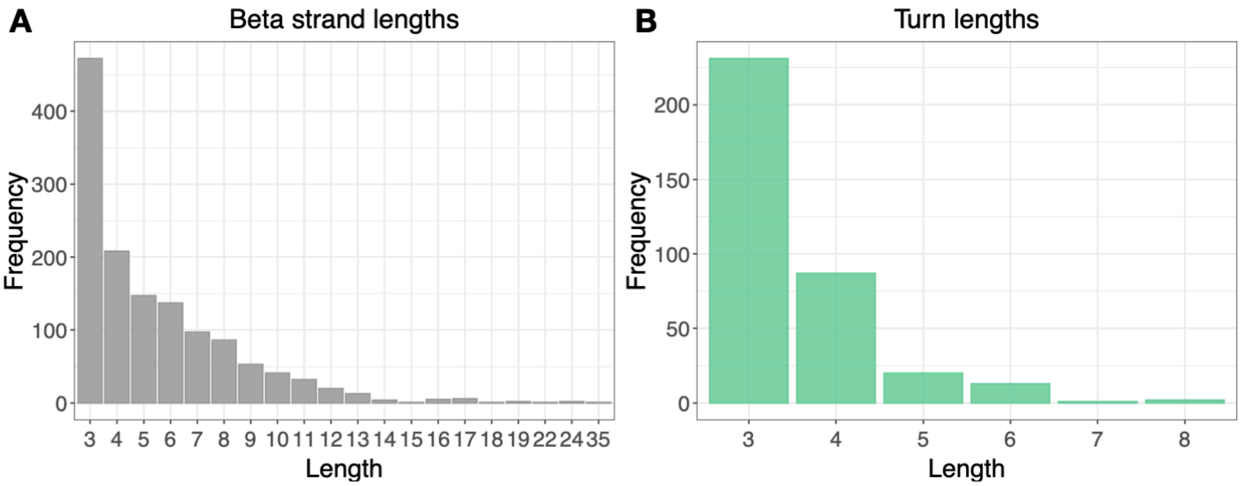

Supplementary Figure 5 Secondary structure lengths of resolved amyloids. We downloaded data from the WALTZ-DB data portal, filtered for amyloids, then manually parsed the Uniprot entries of each sequence to obtain the distribution of (A) beta strands and (B) turns across 80 sequences.

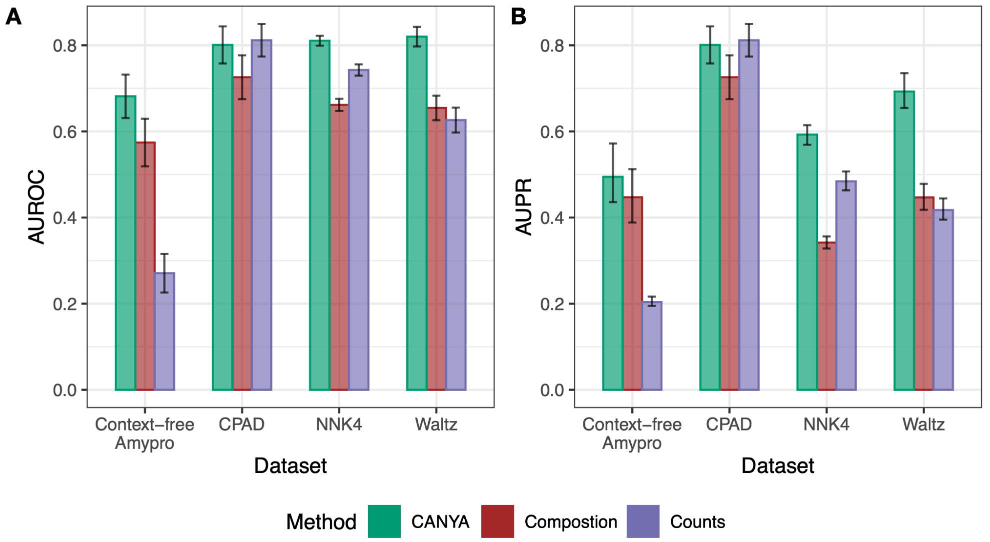

Supplementary Figure 6 The performance of CANYA compared to simpler models. Area under the receiver-operating characteristic and precision-recall curves (AUROC, AUPR respectively) of each method on a corresponding testing set (Methods, Supplementary Table 6). “Composition” corresponds to training a simple logistic regression model using amino acid composition (between 0.0 and 1.0, proportions) over the training NNK dataset, and “Counts” corresponds to training the same model with raw, unnormalized amino acid counts (between 0 and 20, integer values).

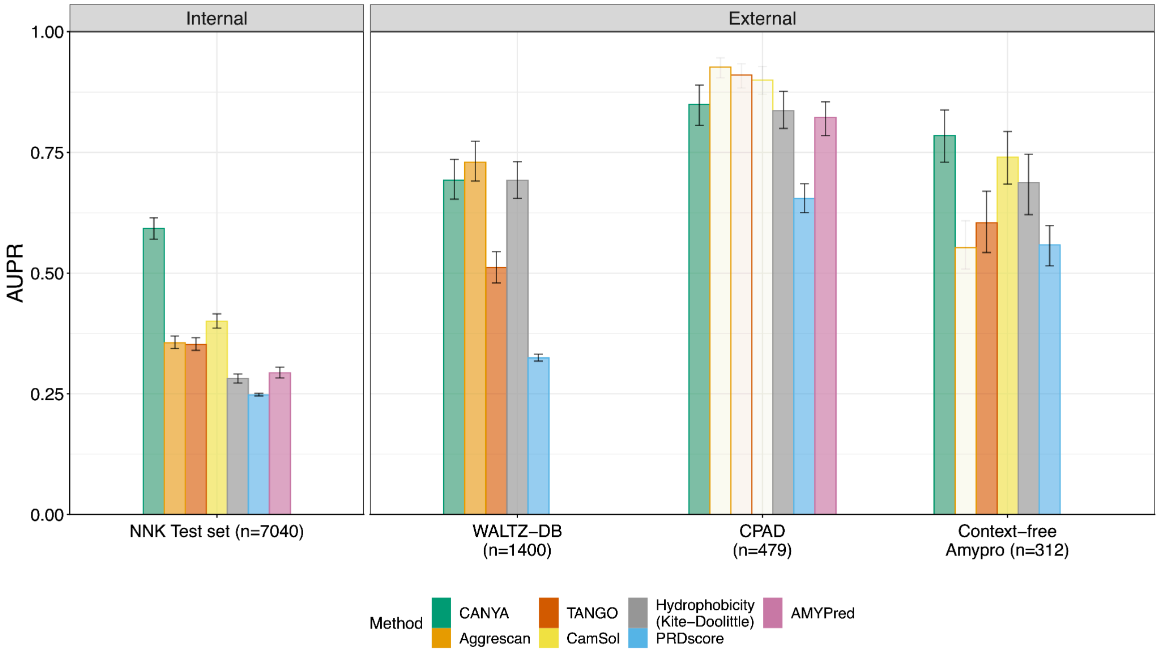

Supplementary Figure 7 The performance of CANYA compared to previous approaches on testing datasets. Area under the precision-recall curve (AUPR) of each method on a corresponding testing set. Low-opacity bars represent cases in which the method used data from the testing dataset to do its training, and thus are not valid out-of-sample evaluations. See text for additional descriptions of datasets (Methods, Supplementary Table 6).

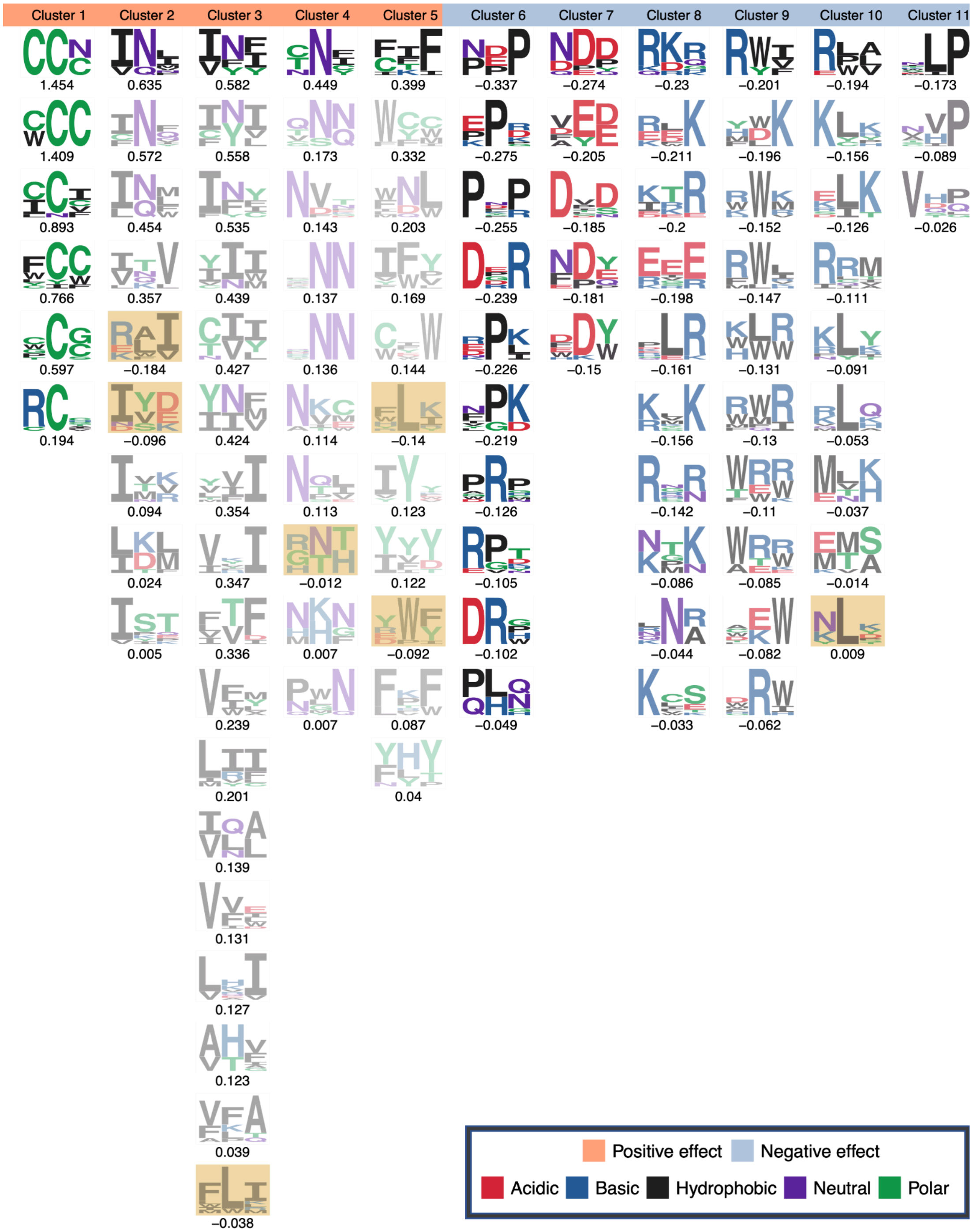

Supplementary Figure 8 Physicochemical motifs discovered by CANYA prior to performing quality control. We removed filters from clusters if their GIA effect direction was opposite the sign of the effect of the strongest filter. We highlight which motifs were excluded from downstream xAI analysis in yellow.

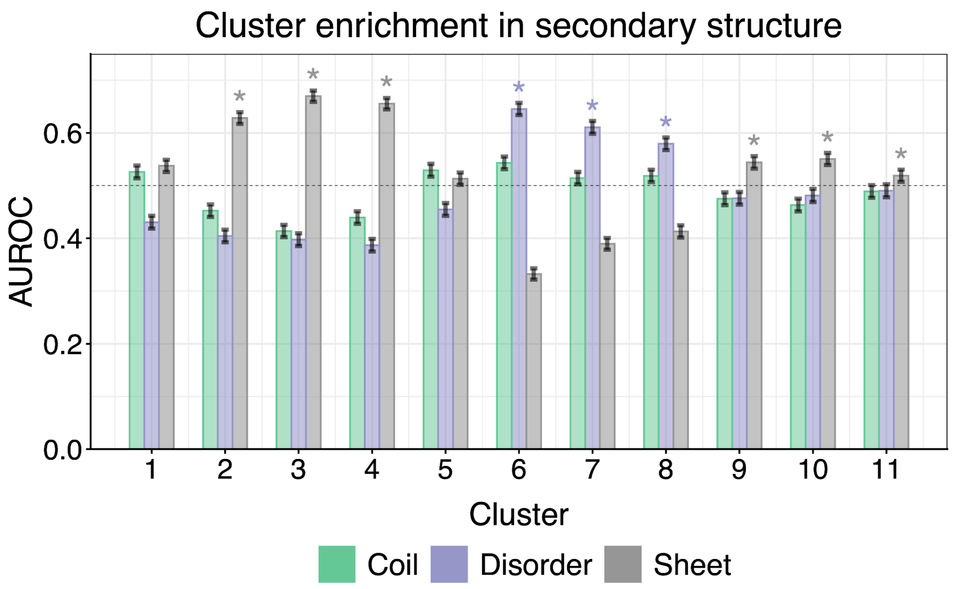

Supplementary Figure 9 Secondary structure enrichment of motifs discovered by CANYA. We collected sequences from the StAmP dataset then collected their convolution layer activation energies from CANYA. Across all sequences, we examined whether a specific cluster had higher activation (pattern matching) within a specific secondary structure by calculating the AUC between the activation energy on a specific secondary structure (Methods). Asterisks represent structures for which the enrichment was significantly higher than both 0.50 and the second most-enriched structure.

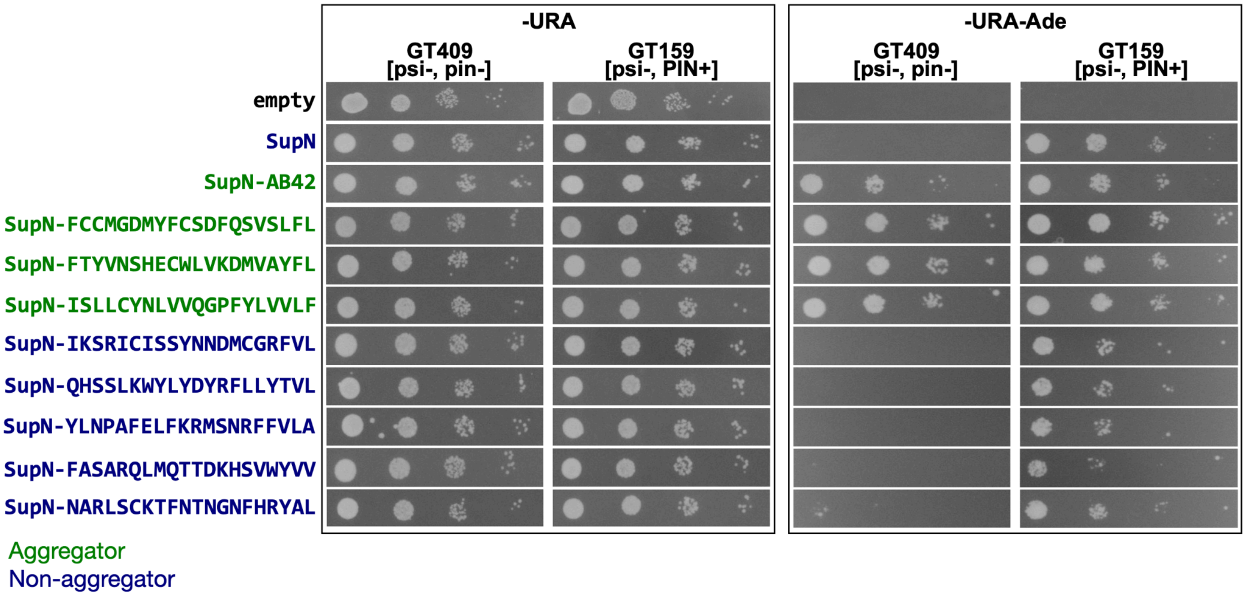

Supplementary Figure 10 Evaluating the expression and aggregation of sequences with hydrophobic C-termini. After *in silico* experiments revealed that many motifs contain dampened effects toward the C-terminus of the construct, we evaluated whether this may be due to a bias in our assay. Many of the motifs with large effect sizes contained hydrophobic residues, and their effects tended to wane toward the C-terminus. Nonetheless, hydrophobic C-termini may lead to reduced expression of a sequence, which may potentially confound the aggregation score by artificially deflating it. We therefore performed an experiment across two cell-lines. In the yeast strain GT409, employed for the high-throughput selection assays, the amyloid aggregation propensity of sequences fused to Sup35N determines the conversion of Sup35 into aggregates, conferring cells the ability to grow without adenine, commonly referred to in prion biology as a change from the [psi-] to the [PSI+] phenotype. In a different strain, GT159[26], the background presence of [PIN+] aggregates facilitates the conversion of Sup35N into aggregates, even when it is not fused to amyloid sequences. The ability of GT159 cells to grow without adenine thus depends exclusively on the expression of Sup35N. We therefore selected 8 sequences with high hydrophobicity indices at the C-terminus, covering both aggregators and non-aggregators, and evaluated the growth in selective conditions (-URA-Ade, right panel) of GT159 cells expressing them fused to Sup35N as a way to probe whether the Sup35N fusions were expressed and not degraded. All sequences sustained growth of the GT159 cells in selective conditions, suggesting no major differences in expression for these sequences. In parallel, to confirm their ability to aggregate, we also expressed the same sequences in the GT409 strain employed for the competition experiments and confirmed that only aggregators grow in selective conditions. As a control, cells were also grown in non-selective conditions (-URA, left panel).

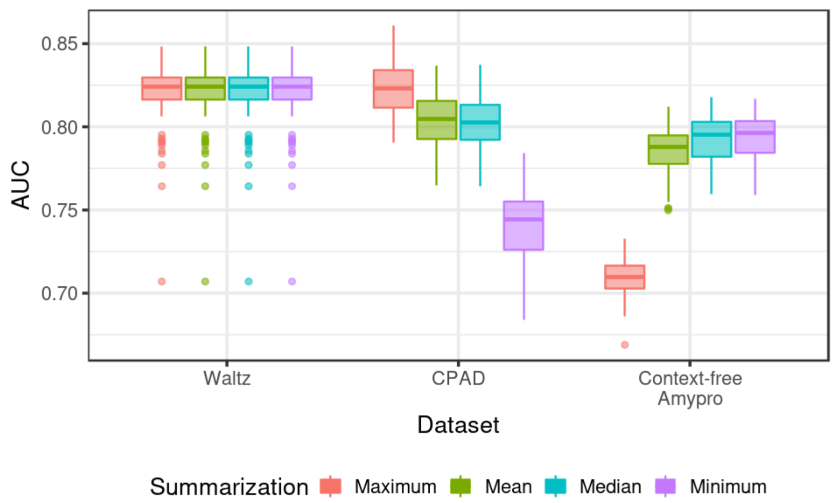

Supplementary Figure 11 The effect of CANYA summarization function on performance. We examined the effect of taking the minimum, maximum, median, or mean CANYA score calculated over all test sequences. Notably, summarizing the CANYA score only affects sequences with length greater than CANYA's input window of 20 residues.

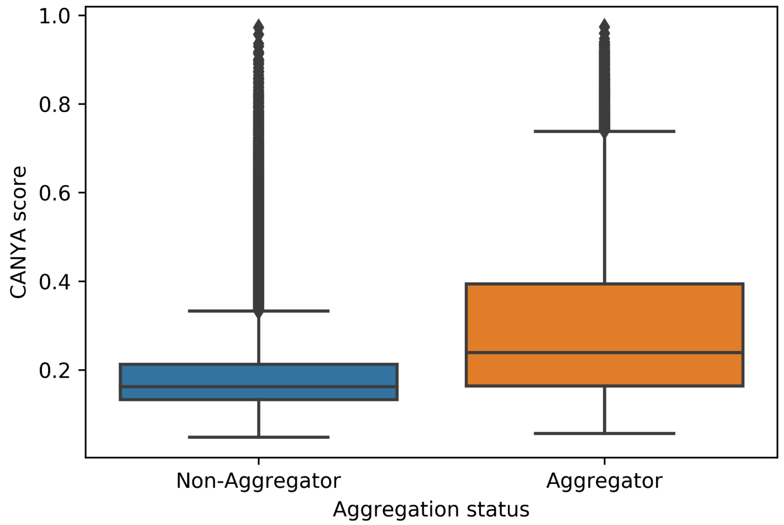

Supplementary Figure 12 Distribution of CANYA scores across training sequences. The distribution of output scores for non-aggregators (n=79,910) and aggregators (n=20,826).

Supplementary Data

Supplementary Data File 1 All sequences recorded spanning each experiment with reported fitnesses, error, and aggregation status
Supplementary Data File 2 Sequences used to train and test CANYA
Supplementary Data File 3 Sequences used in replication experiments with their original measured fitness and fitness from the replication experiment
Supplementary Data File 4 Validation sequences and their corresponding nucleotide sequences
Supplementary Data File 5 Oligo pool and primer sequences for the NNK experiments
Supplementary Data File 6 Read counts and distributions across NNK experiments
Supplementary Data File 7 Transformants measured across each experiment

Supplementary Tables

Supplementary Table 1 Characteristics of external datasets.
Supplementary Table 2 Amino acid frequencies in aggregating and non-aggregating sequences.
Supplementary Table 3 Known human amyloids used in Figure 2 analysis.
Supplementary Table 4 Amino acid composition non-aggregators differently predicted by CamSol and CANYA.
Supplementary Table 5 Amino acid composition of aggregators differently predicted by CamSol and CANYA.
Supplementary Table 6 Filters organized by cluster and effect sizes.
Supplementary Table 7 Amyloid strains used to examine secondary structure enrichment.
